# Release of frustration drives corneal amyloid disaggregation by brain chaperone

**DOI:** 10.1038/s42003-023-04725-1

**Published:** 2023-03-30

**Authors:** Jia Yi Kimberly Low, Xiangyan Shi, Venkatraman Anandalakshmi, Dawn Neo, Gary Swee Lim Peh, Siew Kwan Koh, Lei Zhou, M. K. Abdul Rahim, Ketti Boo, JiaXuan Lee, Harini Mohanram, Reema Alag, Yuguang Mu, Jodhbir S. Mehta, Konstantin Pervushin

**Affiliations:** 1 School of Biological Sciences, Nanyang Technological University, Singapore, 637551 Singapore; 2 Department of Biology, Shenzhen MSU-BIT University, 518172 Shenzhen, China; 3 Singapore Eye Research Institute, 11 Third Hospital Avenue, Singapore, 168751 Singapore; 4 School of Optometry, Department of Applied Biology and Chemical Technology, Research Centre for SHARP Vision (RCSV), The Hong Kong Polytechnic University, Hong Kong, China; 5 Centre for Eye and Vision Research (CEVR), 17W Hong Kong Science Park, Hong Kong, China; 6 Ophthalmology and Visual Sciences Academic Clinical Program, Duke-NUS Graduate Medical School, Singapore, 169857 Singapore; 7 Singapore National Eye Centre, 11 Third Hospital Avenue, Singapore, 168751 Singapore

**Keywords:** Chaperones, Solid-state NMR, Cryoelectron microscopy, Protein aggregation

## Abstract

TGFBI-related corneal dystrophy (CD) is characterized by the accumulation of insoluble protein deposits in the corneal tissues, eventually leading to progressive corneal opacity. Here we show that ATP-independent amyloid-β chaperone L-PGDS can effectively disaggregate corneal amyloids in surgically excised human cornea of TGFBI-CD patients and release trapped amyloid hallmark proteins. Since the mechanism of amyloid disassembly by ATP-independent chaperones is unknown, we reconstructed atomic models of the amyloids self-assembled from TGFBIp-derived peptides and their complex with L-PGDS using cryo-EM and NMR. We show that L-PGDS specifically recognizes structurally frustrated regions in the amyloids and releases those frustrations. The released free energy increases the chaperone’s binding affinity to amyloids, resulting in local restructuring and breakage of amyloids to protofibrils. Our mechanistic model provides insights into the alternative source of energy utilized by ATP-independent disaggregases and highlights the possibility of using these chaperones as treatment strategies for different types of amyloid-related diseases.

## Abstract (web summary)

ATP-independent amyloid-β chaperone L-PGDS disaggregates corneal amyloids in surgically excised human cornea of TGFBI-related corneal dystrophy patients by recognizing and releasing structurally frustrated regions.

## Introduction

The formation of amyloid deposits in different types of tissues is implicated in many degenerative disorders, such as Alzheimer’s disease (AD) and corneal dystrophy (CD). Transforming growth beta-induced (TGFBI)-related CD is the most common heritable stromal CD worldwide^1^. It is characterized by the accumulation of insoluble protein deposits in the corneal tissues, eventually leading to the progressive loss of corneal transparency in patients and blindness^2^. To date, there are 74 different mutations reported in the *TGFBI* gene. Mutations in the *TGFBI* gene results in the mutant protein product, TGFBI protein (TGFBIp) which is proteolytically cleaved in the corneal tissues^3–5^. From our previous proteomics analysis and spatial high-resolution mass spectrometry imaging, we concluded that the TGFBIp is the main component of the amyloid aggregates found in patients’cornea and the TGFBIp-derived peptides are the predominant component of the TGFBIp fibril found in the amyloid deposits, respectively^6,7^. In addition, the genotypes of the *TGFBI* gene are linked to the different isoforms of the amyloid deposits observed in patients^8–10^. Therefore, we hypothesis the fibrils formed by TGFBI-derived peptides with different mutations would display different fibril morphology. Currently, none of the atomic resolution structure of TGFBI fibrils or fibril derived from truncated TGFBIp sequences has been resolved. Biophysical study of different mutants of the 611-633 region of the TGFBIp has revealed that the G623R mutant showed the highest amyloid aggregation propensity and fibril stability^11^.

Polymorphic amyloid degeneration is another form of the amyloid deposit-related corneal disorder that is non-inheritable. This non-inheritable trait of the disease shows that environmental and other factors besides genetics can be associated with corneal amyloid deposition^12,13^. This phenomenon is also observed in Alzheimer’s disease (AD), a genetically complex disorder. It is associated with well-defined risk loci, notably the APOE*4 allele^14^, as well as various environmental factors such as air pollution and bacterial infection^15–17^. This evidence indicates that the occurrence of corneal amyloids might be more widespread than the current estimated number based on population genotyping, warranting urgent attention for both prevention and treatment strategies against corneal amyloid-related diseases.

Most of the amyloid deposits in these corneal diseases are found in the central area of the cornea where the blood supply is limited^18^. Therefore, it is suggested that molecular factors such as protein chaperones found in the blood vessels might play a role in preventing the accumulation of amyloid deposits in the corneal tissues^9^. Tear and aqueous humor primarily nourish the corneal tissue and they contain several native chaperones such as HtrA1 and clusterin^19,20^. However, the protecting effect might not be sufficient to clear amyloid deposits in an adverse genetic or environmental background^9^. Conversely, the cerebrospinal fluid (CSF) has evolved to have a unique set of chaperones to deal with polymorphic amyloid deposits such as Aβ amyloids^21^. Since most amyloid deposits are found in the extracellular region where the amount of ATP is low, the majority of the amyloid chaperones in the CSF are ATP independent^22,23^. Lipocalin-type prostaglandin D synthase (L-PGDS) is the second most abundant protein in the CSF and one of the critical chaperones present in the CSF but is absent in the corneal tissues^22,24,25^ L-PGDS exerts both inhibitory and amyloid disaggregation effects by directly binding to the monomeric Aβ peptides and preformed amyloid fibrils^22,26^. Even though the catalytic activity of L-PGDS to isomerize Prostaglandin H2 depends on its C65 residue, we have shown that the C65A mutant of L-PGDS retained its chaperone activity in our earlier study^22^.

Furthermore, L-PGDS can effectively penetrate the blood-brain barrier and is potentially an effective drug delivery vehicle^27,28^. These properties of L-PGDS suggest that it can reach the stromal layers in the corneal tissues when applied topically. Indeed, here we show that the application of L-PGDS clears amyloid aggregates from surgically excised human cornea and releases hallmark proteins typically trapped in amyloids^22^. Since the molecular mechanism of amyloid disaggregation by L-PGDS and other ATP-independent chaperones remains unknown, we report atomic structural models of an amyloid fibril produced with TGFBIp-derived peptides associated with CD and its complex with L-PGDS obtained by cryo-electron microscopy (cryo-EM) and solid-state NMR (ssNMR). Together with subsequent mutagenesis experiments and MD simulations, the chaperone is revealed to specifically recognize and release structural frustrations in amyloids. This release of frustration creates a local mechanical defect in the amyloid mesoscopic order. This disaggregation mechanism appears to be distinctly different from the entropic pull of ATP-dependent Hsp70 chaperone^29,30^ and the bottle brush effect of the long alkyl chain detergents^31^. The salient feature of the proposed mechanism, the release of local structural frustrations in both amyloid and chaperone, might be a source of free energy of binding. This energy enables the chaperone to scan the complex environment of the amyloid deposits and with higher specificity, recognize pathological fibrils rich in structural frustrations. We show that ATP-independent chaperones might effectively bind, locally restructure, and disassemble the diverse and polymorphic TGFBIp-derived amyloids to protofilaments, thus clearing the affected corneal tissues. This unusual application of a brain-abundant chaperone in the cornea may represent an attractive therapeutic avenue in treating TGFBI-related CD and potentially other amyloid-related conditions.

## Results

### Atomic model of TGFBIp G623R fibrils

The peptide (^611^EPVAEPDIMATNGVVHVITNVLQ^633^) is one of the most amyloidogenic proteolytic fragments of the 4^th^ FAS-1 domain of TGFBIp associated with TGFBI-related CD^11,32^. Since this 611-633 region with the G623R mutation of the TGFBIp showed the most microscopically homogeneous fibrils and well-resolved ssNMR spectra^11^, we have chosen it as our primary model system for structural studies.

**Fig. 1:**
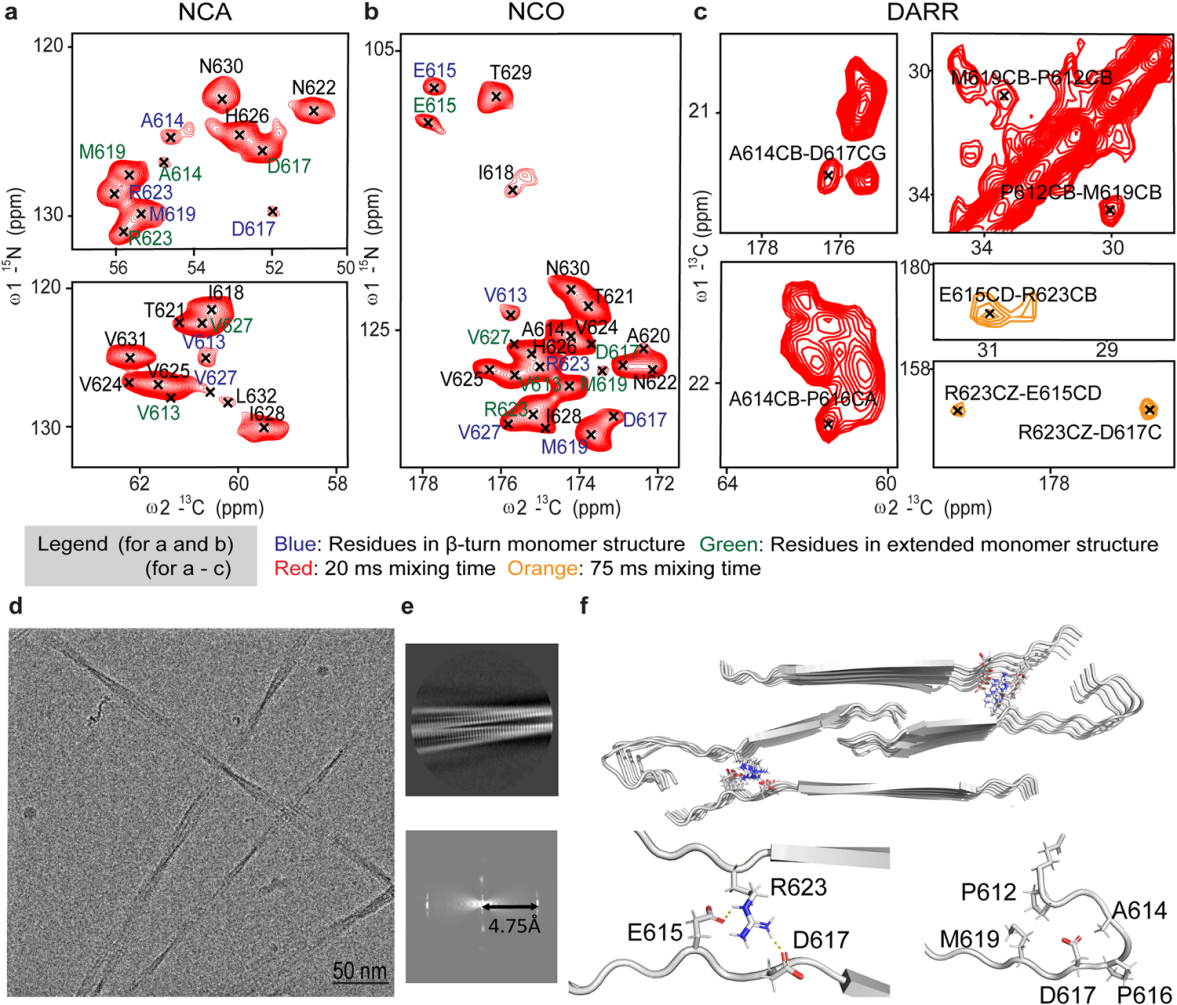
Atomic model of the G623R fibril reconstructed using ssNMR and cryo-EM. **a** 2D NCA and (**b**) 2D NCO (Contour level: 7e + 006, 37, 1.1) (**c**) 2D ^13^C-^13^C DARR with contact time 20 ms and 75 ms spectra of the ^13^C-^15^N labeled G623R fibril represented in red and orange, respectively. Residues coming from the extended conformation and β turn regions are colored in blue and green, respectively. (Contour level: 1.5e + 006, 37, 1.1). **d** Representative cryo-EM image of the G623R fibril. **e** 2D class average of the fibril and its respective power spectrum showing distance between meridian line and 1^st^ layer line of 4.75 Å. **f** Calculated model of G623R fibril comprising 5 repeating units each consisting of 4 monomers.

Uniformly ^13^C- and ^15^N-labeled G623R and A620D variants of the peptide strongly associated with TGFBI-related CD phenotype were expressed in *E.coli* using specifically developed liquid-liquid phase separating BEAK tag protein^33^. The fusion protein condensates prevent the chemical modifications of the peptides during expression and result in the reproducible formation of structurally homogeneous fibrils (Fig. S1). Backbone ssNMR such as NCA, NCO, DARR, NCACX, NCOCX and CANCO (Fig. 1 and S2–3) were used for complete resonance assignment and collecting of structural constraints. Fibrils formed with G623R and A620D variants exhibited well-resolved NCA/NCO and NCA spectra shown in Fig. 1a-b and Fig. S4, respectively. However, A620D showed resonance doubling and tripling found in residues indicating structurally polymorphic fibrils when compared to G623R fibrils (Fig. S4). This prompted us to proceed high-resolution structural studies with G623R fibrils. Fig 1a, b show excerpts from the ^13^C-^15^N 2D NCO and NCA spectra of G623R fibrils with the residue-specific assignment (see Table S1 for the complete assignment), respectively. Notably, V613, A614, E615, D617, M619, R623, and V627 exhibit two sets of cross-peaks assigned to individual residues. The 2D ^13^C-^13^C DARR spectrum with a 20 ms dipolar contact period shows alternative ^13^C-^13^C dipolar contacts between residues P612 and M619 and A614 and D617 (Fig. 1c). These data suggest two conformations with one consists of β-turn and another, an extended monomer structure at the N-terminus in the fibril context. The presence of β-turn is further supported by comparing the difference in chemical shifts between β-turn and the extended form of the peptides using the online chemical shift prediction platform (ProShift)^34^ (Table S2). From the chemical shift analysis, residues V613, A614, E615, D617, M619, R623 and V627, found in the β-turn exhibited a significant difference in the chemical shift of the nitrogen atom as compared to the extended peptide. In addition, in the 2D DARR spectrum with the longer dipolar contact time (75 ms), contacts between E615, D617, and R623 are observed (Fig. 1c). These long-range contacts could indicate inter-strand interaction within the fibril. We then utilized the TALOS+ software to predict the backbone ϕ and ψ torsion angles (Table S3) and secondary structure (Fig. S5). Finally, we included the distance and the torsion angle restraints calculated from the chemical shifts in the structure calculation of the monomer in CYANA^35^ (Tables S1, S3) (Table 1).

**Table 1:** NMR and refinement statistics for protein structures.

1 repeating unit (4 monomers) PDB code: 8HGA BMRB ID: 36518	
*NMR distance and dihedral constraints*	
Distance constraints	
Total NOE	1032
Intra-residue	972
Inter-residue	56
Sequential (|*i* – *j* | = 1)	12
Long-range (intermolecular)	44
Hydrogen bonds	0
Total dihedral angle restraints	168
ϕ	84
ψ	84
*Structure statistics*	
Violations (mean and s.d.)	
Distance constraints (Å)	0
Dihedral angle constraints (°)	0
Max. dihedral angle violation (°)	0.09
Max. distance constraint violation (Å)	0.25
Deviations from idealized geometry	
Bond lengths (Å)	1
Bond angles (°)	1
Impropers (°)	2
Average pairwise r.m.s. deviation was calculated among 20 structures** (Å)	
Heavy (from residues 11 to 20 central molecules in final structure bundle)	2.4
Backbone (from residues 11 to 20 central molecules in final structure bundle)	0.9

We used a hybrid structure determination approach combining ssNMR and cryo-EM to produce the atomic resolution model of the fibril. Figure 1d, e shows the representative image of the EM micrographs and the 2D class averages obtained from the cryo-EM images. Next, we obtained an electron density map of the fibrils with 4.9 Å resolution after 3D refinement (Fig. S6b, c) (Table 2). Finally, we obtained the atomic model of the G623R fibrils through the combination of the data obtained from both techniques (Fig. 1f). From the analysis of the G623R fibril model, the R623 residue is involved in the formation of intermolecular salt-bridge with D617 and E615 in the opposite strand (Fig. 1f). This is also consistent with the dipolar contacts between these residues observed in the ^13^C-^13^C DARR and the NCO spectra (Fig. 1c and S7). Furthermore, when we simultaneously swapped the residues participating in this salt bridge in the double mutant (R623D, D617R), uniform fibrils could again be produced (Fig. S8), thus validating the presence of this salt bridge in the amyloid model. Additionally, this thermodynamically favorable interaction provided by the inter-strand salt bridge likely accelerates the fibril growth of the G623R peptide compared to wild-type and other mutants^11^. In the fibrils of A620D where the salt bridge is absent, a significant larger degree of polymorphs was observed, pointing to a stabilizing role of this salt bridge (Fig.S4).

**Table 2:** Cryo-EM data collection, refinement and validation statistics.

	TGFBIp G623R fibril (EMDB-34813) (PDB 8HIA)
*Data collection and processing*	
Magnification	165 kx
Voltage (kV)	300
Electron exposure (e–/Å^2^)	40
Defocus range (μm)	−3 to −1
Pixel size (Å)	0.85
Symmetry imposed	2_1_
Initial particle images (no.)	1711242
Final particle images (no.)	48522
Map resolution (Å)	4.9
FSC threshold	
Map resolution range (Å)	1.7–5.1
*Refinement*	
Initial model used (PDB code)	–
Model resolution (Å)	4.9
FSC threshold	
Crossover distance	120 nm
Fibril Width	20 nm
Model resolution range (Å)	510.0–1.7
Map sharpening *B* factor (Å^2^)	−98.95
Model composition	
Non-hydrogen atoms	3540
Protein residues	460
Ligands	–
*B* factors (Å^2^)	
Protein	–
Ligand	
R.m.s. deviations	
Bond lengths (Å)	0.001
Bond angles (°)	0.328
Validation	
MolProbity score	2.89
Clashscore	1.11
Poor rotamers (%)	2.86
Ramachandran plot	
Favored (%)	87.62
Allowed (%)	12.14
Disallowed (%)	0

### Interaction between L-PGDS and G623R fibril

**Fig. 2:**
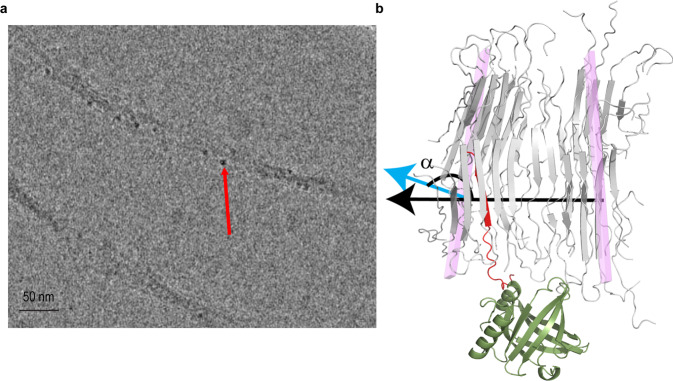
Binding of L-PGDS to the G623R fibril. **a** Representative cryo-EM image of the G623R fibril and L-PGDS conjugated with heme for contrast enhancement. The red arrow shows the localization of L-PGDS along the convex surface of the fibril. **b** Snapshot of MD simulation of the G623R fibril (light gray) and L-PGDS (olive green) complex at time step 6 ns before the opening of the salt bridge (E615 and R623). The blue and black arrows represent the normal of the plane of 2nd and 9th monomer defined by CA of A620, N622 and H626, respectively. The angle α is formed between the normal of the 2^nd^ and 9^th^ planes. The interacting monomer is highlighted in red.

A remarkable feature of L-PGDS is its ability to coordinate two iron heme molecules with picomolar affinity while retaining its Aβ chaperone function^36^. Since the L-PGDS molecule is too small to be directly imaged in cryo-EM, here we utilized the L-PGDS-heme complex to visualize the binding locations of L-PGDS on the G623R fibril using bound heme as a contrast enhancer. Both heme loaded and free L-PGDS can effectively chaperone amyloids as shown in a previous study^36^. In high-resolution cryo-EM micrographs, L-PGDS-heme complexes appear as electron-dense spots with a size corresponding to that of L-PGDS. They are located on the convex side of the bent G623R fibril (Fig. 2a). The stoichiometry directly calculated from the cryo-EM images, 1:7 (LPGDS: peptide), closely corresponds to mixing stoichiometry of 1:10 indicating that majority of L-PGDS are bound to the fibrils (Fig. 2a). An analysis of cryo-EM micrographs of fibrils in complex with L-PGDS indicates that bending of the fibrils, an observation notably only in the presence of L-PGDS, is accompanied by a 4-fold reduction in the persistence length from 12500 nm down to 3300 nm and considerable shortening of the fibril contour length (Fig. S9) as the direct consequence of L-PGDS binding.

**Fig. 3:**
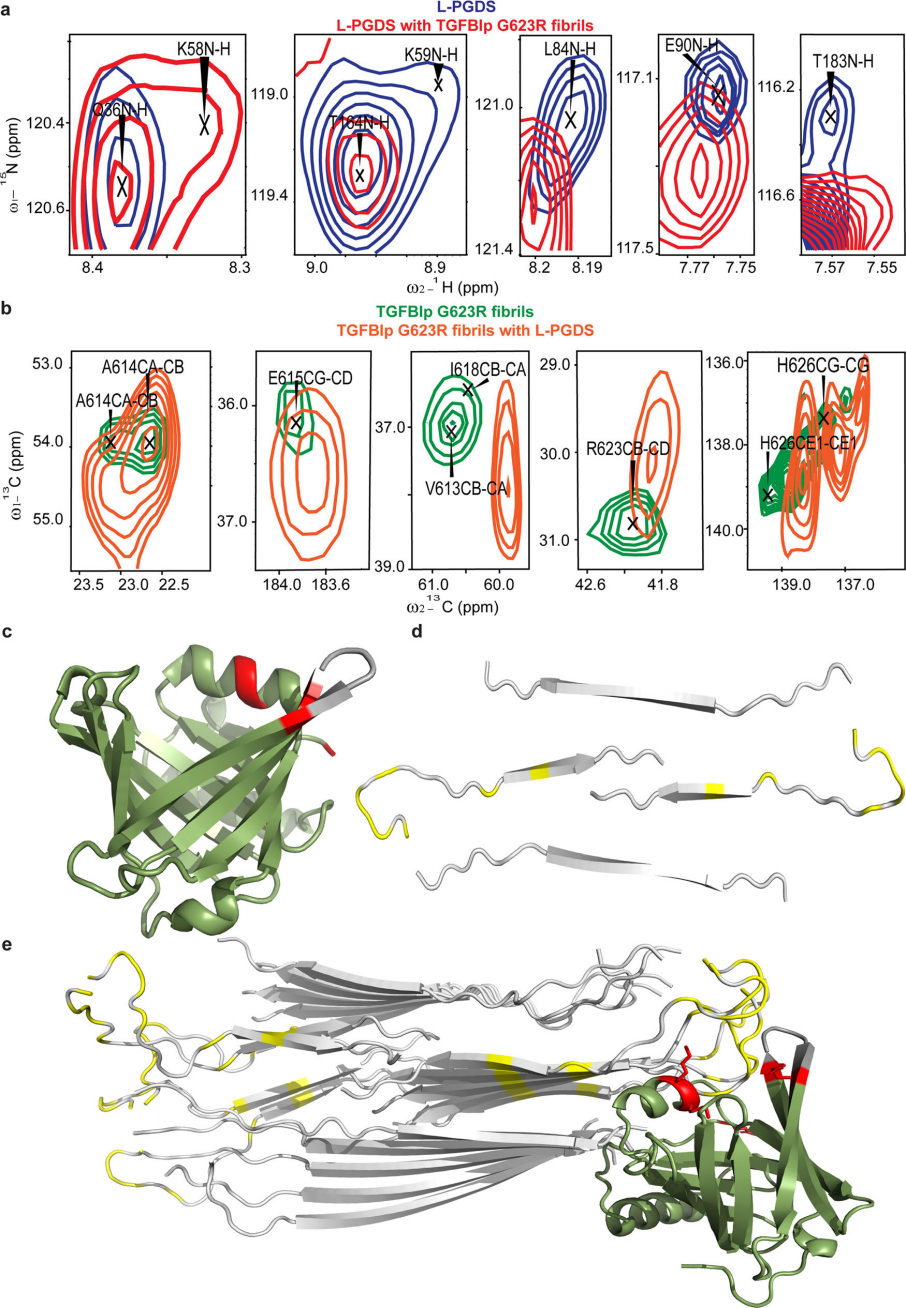
Mapping of interactions between G623R fibril and L-PGDS. **a** Selected residues of ^1^H-^15^N HSQC spectra of the ^15^N-labeled L-PGDS in the absence (blue) and presence of G623R fibrils (red). **b** ^13^C-^13^C DARR spectra of the ^13^C and ^15^N-labeled G623R fibrils in the absence (green) and presence of L-PGDS (orange). **c**, **d** Residues of L-PGDS and G623R fibril, respectively, exhibiting significant chemical shift changes as highlighted in red and yellow. **e** NMR data-guided docking model of G623R fibril and L-PGDS (PDB code: 4IMN)^3^.

Next, the details of interactions between L-PGDS and G623R fibrils at molecular level were ascertained with solution and ssNMR. Firstly, ^15^N-HSQC solution NMR spectra was obtained for ^15^N labeled L-PGDS in the absence and presence of G623R fibril to identify potential fibril-binding sites and map the residues on L-PGDS. We observed significant chemical shift perturbation in residues K58, K59, L84, E90, and T183 of L-PGDS when the fibrils were added (Fig. 3a, S10a, b). These residues are highlighted in red on the crystal structure of L-PGDS^37^ (Fig. 3c). In addition, cross peaks from residues D37, A60, L84, R85, K86, E90, T91, G135-A146, A157 and T183 showed a significant reduction in the signal intensity (Fig. S10c). Residues that are affected by the presence of L-PGDS seems to cluster around the top of the calyx which is an area enriched with basic residues such as K and R, possibly suggesting the presence of a positively charged binding groove in L-PGDS for interaction. To confirm our hypothesis, we also examined the residues in the G623R fibrils involved via ssNMR spectroscopy to map the potential L-PGDS binding site on the fibril. We recorded the 1D ^13^C CP and 2D ^13^C-^13^C DARR spectra of the L-PGDS and G623R fibril complex (Fig. 3b, S11). The binding of L-PGDS on the fibril caused a significant change in chemical shifts of E611, V613, A614, E615, D617, R623, and H626 of the fibril, as shown in Fig. 3b and S11. The affected residues are localized to the β-turn conformation of the monomers (Fig. 3d), suggesting that L-PGDS might bind from the side of the fibril as observed in cryo-EM micrographs (Fig. 2a). Subsequently, we obtained a docking model of the interaction between L-PGDS and G623R fibrils guided by the NMR chemical shift perturbations using the online protein docking platform HADDOCK^38^ (Fig. 3e). In line with our findings, the model exhibits the complementary fitting of the negatively charged β-turn region of the fibril into the positively charged binding groove identified in L-PGDS. The residues in the helical region of L-PGDS interact with the β-sheet strands of the fibril through helix-β strand interaction^39^ while the basic residues in the loop region of L-PGDS interact with the β-turn regions of the fibril.

**Fig. 4:**
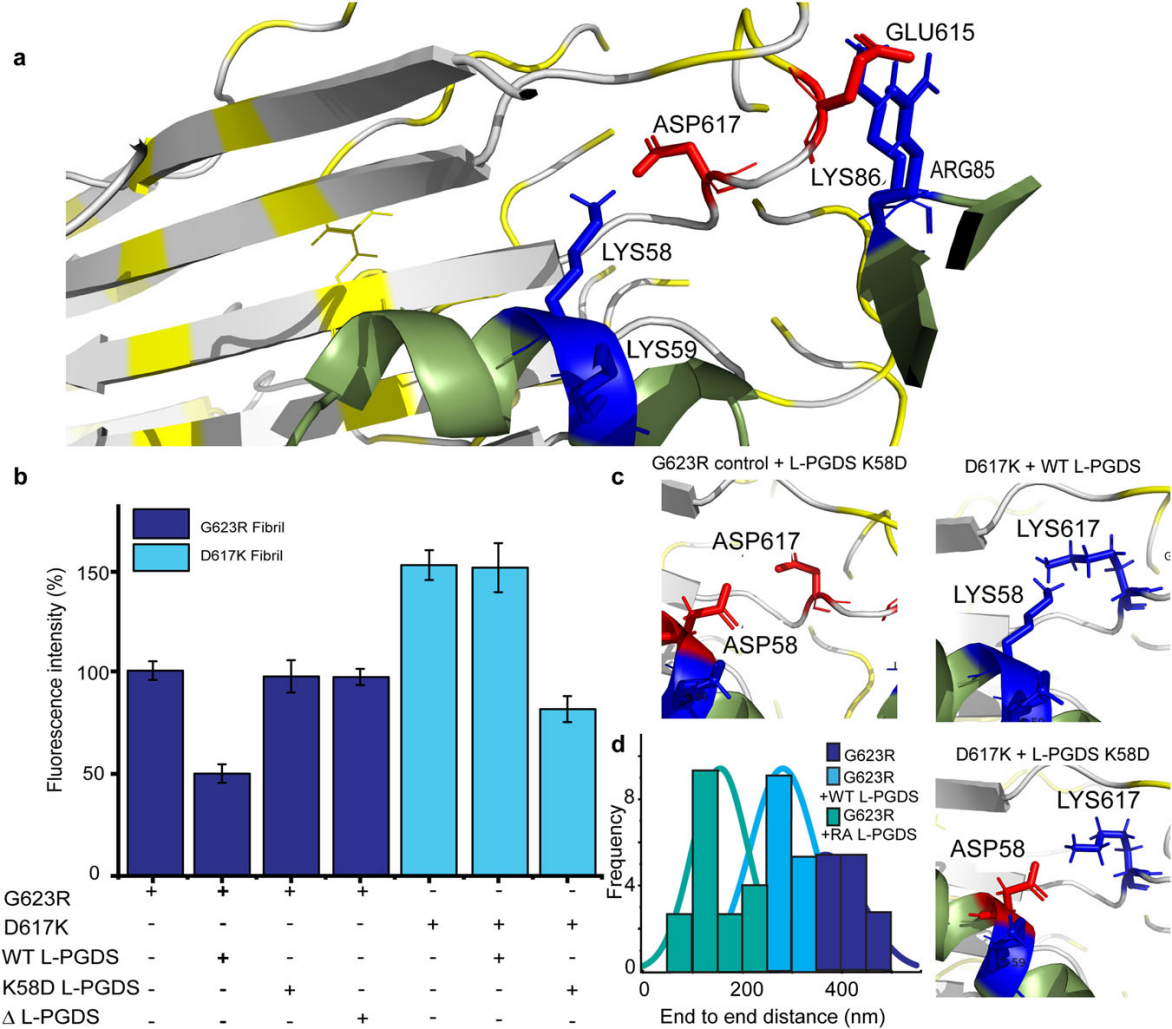
Interaction interface between L-PGDS and the G623R fibril. **a** Zoomed-in image of the docking model displaying electrostatic interaction between E615, D617 of G623R fibril and K58, K59 & R85, K86 of L-PGDS, respectively. **b** End point ThT assay showing the ThT fluorescence intensity of the G623R and D619K fibril, its complex with L-PGDS, mutant K58D of L-PGDS, and mutant K58A, K58A of L-PGDS (ΔL-PGDS) *n*  ≥  3 independent experiments. **c** Zoomed-in 3D representations of the interactions between G623R with mutant K58D of L-PGDS, mutant D617K of G623R with L-PGDS, mutant D617K of G623R with mutant K58D of L-PGDS. The negatively charged ASP residue is colored in red and the positively charged LYS residue is colored in blue. **d** Histogram showing the end-to-end distance of G623R fibril incubated with PBS buffer only, L-PGDS and double mutant (K58R, C65A) of L-PGDS (RA L-PGDS).

We confirm our reconstructed L-PGDS/fibril model (Fig. 4a) by a series of site-directed mutations of L-PGDS and the TGFBIp-derived peptide. First, we produced a double Lys knockout L-PGDS mutant (ΔL-PGDS) where both fibril interacting residues K57 and K58 were substituted to Ala. After 24 h of incubation with preformed G623R fibrils, ΔL-PGDS showed a reduction of less than 2% in ThT intensity. In contrast, the WT L-PGDS exhibited a reduction of ~30% as compared to the control G623R fibrils (Fig. 4b). Reverse mutations of an identified interacting pair oppositely charged amino acids, K58 in L-PGDS and D617 in G623R (thereby referred to as D617K) fibrils, also further verified the key role of electrostatic interactions in the recognition and disaggregation of L-PGDS. Only when both residues in L-PGDS and G623R were mutated and the charges were complementary, as shown in Fig. 4a, the amyloid disaggregation activity was restored in the endpoint ThT assay (Fig. 4b, c).

Since the primary effect of the L-PGDS interaction with the fibrils is the reduction in the fibril’s end-to-end length, we used this parameter to compare the disaggregation efficiency of the G623R fibrils by various L-PGDS mutants. We found that the substitution of K58 with arginine and the catalytic C65 with alanine (RA L-PGDS) in L-PGDS disaggregates the G623R fibrils into significantly shorter protofibrils (Fig. 4d and Fig. S12). This observation further validates the proposed structural model and indicates that direct mutagenesis can further enhance the disaggregation effect of L-PGDS.

### Release of fibrils’ structural frustration

We identified the structurally frustrated interactions within the G623R fibril using the online server, Frustratometer^40^. From Fig. S13a, b the highly frustrated regions within the G623R fibrils, represented by red lines, are clustered around the β-turn region of the fibril. Table S4 highlights the type of interaction, frustration energy, frustration index, and frustration state of the residue pairs. The observation of frustrated regions in the β-turn region of the G623R fibril agrees with one of the three leading causes of frustration suggested by Sawaya et al.^41^. The β-turn region is enriched with negatively charged residues (E615 and D617) stacked upon each other which experiences strong charge-charge repulsion. It is also interesting to note that the frustrated regions of the fibril seem to overlap with the L-PGDS binding site. Thus, we proposed that L-PGDS recognizes and binds to the frustrated regions of the fibril through electrostatic interactions as shown in Fig. 4a.

Full atom MD simulations of the G623R fibril fragment, consisting of 10 repeating units of fibrils, with L-PGDS docked to the 5th repeating unit were performed for 50 ns to track changes to the fibril structure upon interaction with L-PGDS (Supplementary data 2). A significant increase in distance between the N-terminal residues (E611 and P616) with respect to the lateral β-sheet region of that monomer (Fig. S14a) was observed, resulting in an extended out-of-register conformation in its N-terminus (Fig. S14b) (Supplementary data 3 and Supplementary video). This local conformational rearrangement essentially releases most of the structurally frustrated interactions associated with the affected monomer (Table S4). The structural model estimates a free energy of −3.7 kcal/mol/monomer due to frustration release (Table S4), which is comparable to the total folding free energy per monomer of the intact fibril. This free energy is primarily used to increase the affinity of L-PGDS to the fibril with subsequent bending of the amyloid at the binding site, as shown in Fig. 2a. This bending can be directly observed along the MD simulation trajectory as the angle between normal vectors to the planes of the 2nd and the 9th repeating units of the fibril (Fig. S14c and S15). A critical increase in the bending angle at the site of L-PGDS binding subsequently results in the breaking of E615 to R623 intermonomer salt bridge, evidenced by the coincidence of the sharp increase in the distance of the residue pair with the increase of bending angle (Fig. S14c, d). Intriguingly, this disruption of the salt bridge is also manifested in the chemical shift perturbations observed for these residues in G623R fibrils after L-PGDS binding.

### Amyloid inhibition and disaggregation by L-PGDS

The ThT-dye mediated fibrillation and disaggregation assay showed L-PGDS concentration-dependent inhibition of G623R fibril growth and disaggregation of the preformed fibrils. L-PGDS at a molar ratio of 1:2 with the G623R peptides exhibits the strongest G623R peptide aggregation inhibition (94%) when compared to the other ratios of 1:5 (89%) and 1:10 (55%) over a continuous period of 48 h (Fig.S16a). The inhibitory effects of L-PGDS of varying molar ratios were visualized using fluorescence microscopy and further confirmed with circular dichroism spectra (Fig. S16b, c). Next, we added L-PGDS in different molar ratios to the preformed fibrils. The plateau in the ThT-dye fluorescence was reached after 40 h of peptide aggregation. Once L-PGDS was added at that time point, we observed a spontaneous decrease in the ThT intensity. After 24 h of incubation with L-PGDS at molar ratios of 1:2, 1:5, and 1:10, the ThT intensity showed a reduction of 52%, 27%, and 19%, respectively, as compared to the control G623R fibrils (Fig. S17a–d). A similar disaggregation progression was observed with L-PGDS applied to the other mutants of TGFBIp peptides such as M619K, A620D, N622H, N622K, R124C, and R124H fibrils (Fig. S18). In summary, L-PGDS exhibited amyloid inhibition and disaggregation abilities at all tested concentrations with varying effectiveness in a concentration-dependent manner. Since L-PGDS is not expressed in corneal tissues, we also examined the toxicity of the WT L-PGDS at different concentrations corresponding to different stoichiometries used in the previous assays on human corneal fibroblasts using live cell imaging. (Fig. S19). The results show that prolonged exposure of the cell culture to L-PGDS at concentrations below 10 µM does not induce significant cytotoxicity.

### Corneal amyloids disaggregation by L-PGDS

**Fig. 5:**
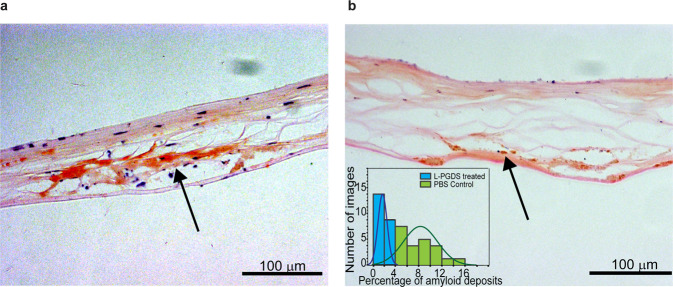
Ex vivo disaggregation effects of L-PGDS on corneal samples. Corneal images collected from a patient with R124C mutation with Congo red staining incubated with (**a**) control PBS buffer and (**b**) the same buffer containing 10 μM L-PGDS. The amyloid deposits are indicated by the black arrow. The histogram shows the distribution of the area of amyloid deposits in corneal samples treated PBS control and 10 μM L-PGDS.

Surgically excised corneal sample obtained from a TGFBI-related CD patient were treated with L-PGDS or control buffer. The sample was subsequently sectioned, stained with Congo red dye, and viewed under a brightfield microscope. We compared the adjacent slices of the corneal samples treated with control buffer and L-PGDS to observe the ex vivo amyloid disaggregation effects of L-PGDS. The corneal sections treated with L-PGDS exhibited significantly smaller areas of Congo red-stained amyloid deposits than the control sections (Fig. 5 and S20). The reduction of the amyloid deposits shows that L-PGDS can break down the corneal amyloids and clear the aggregates in the corneal stromal areas. The key proteins in the corneal amyloids were established by matrix-assisted laser desorption ionization mass spectrometry imaging^7^. In addition to the histological images, proteomics data also showed that the application of L-PGDS releases the amyloid-associated proteins enriched in the amyloid deposits found in the patient’s cornea tissue^6^ such as histone H2B type 1, serum amyloid P component and clusterin as compared to those treated with PBS buffer only (Table S5).

## Discussion

Frustrations within folded functional protein structures are typically minimized by selective evolutionary pressure. However, a certain level of frustration in proteins can be tolerated when they contribute to the protein’s function^42^. CSF-abundant chaperone L-PGDS represents an example of such evolution showing a minimally frustrated calyx-structured core, a significantly frustrated helix (residues 52–60) and a frustrated extended loop (residues 84-89) forming a binding interface with amyloids (Fig. S13c). In contrast, pathological amyloids may escape entirely or be less subjected to selective pressure as many reported amyloids show significant frustrations in their structures^41^. As a result, the pathological amyloid fibrils are more likely to contain frustrated regions and are structurally polymorphic^41,43^. The repetitive stacking nature of amyloid fibrils further magnifies the structural frustration due to charge-charge repulsion, torsion angle frustration, and cavities in structure^41^. We propose that these frustration elements could act as common hotspots for amyloid ATP-independent disaggregases to target the fibrils out of their physiological context. Here, our data shows that the amyloid disaggregation effects of L-PGDS could also apply to amyloids found outside the brain by targeting these shared features in the fibrils implicated in corneal diseases.

The detailed disaggregation mechanism of L-PGDS derived from our data is as follows: First, the frustrated helix (residues 52-60) region of L-PGDS anchors to the lateral surface (side-by-side packing of the β sheets) of the G623R fibril via extensive hydrophobic interaction (helix-β strand interaction)^39^. This surface binding of the frustrated helix to the β-strand is non-specific, thus allowing L-PGDS to recognize and target different types of amyloid clients such as Aβ fibrils^22^ or polymorphic amyloids formed by a variety of proteolytic amyloidogenic peptides from corneal TGFBI protein. This initial binding strategically places the extended loop (residues 84–89) region of L-PGDS near the frustrated β-turn region of the fibril, which is enriched with proximal negatively charged residues (E615 and D617) (Fig. 4a). Next, the electrostatic interaction between the positively charged residues in the loop region of L-PGDS (R85 and K86) and negatively charged residues of the fibril (E615 and D617) (Fig. 4a) and the corresponding flipping out of the N-terminus of the monomer at the β-turn region into the extended-out conformation shown in Fig S13B releases −3.7 kcal/mol/monomer of the frustration-free energy stored within the fibril (Table S4). This free energy is then utilized to increase the affinity of L-PGDS to the fibril and the restructuring of the fibril which results in a mechanical defect in the fibril structure, causing the persistence length of the fibril to decrease by 4-fold (Fig. S9). Persistence length is the mechanical property of a fibril, reflecting its bending rigidity^44,45^. In contrast, the contour length is the end result to the exposure of amyloids to L-PGDS and depends on the exposure time and temperature. At the given temperature, the amplitude of thermal fluctuation would determine the average contour length at the equilibrium and therefore the depth of disaggregation (Fig. S9 and S17). Ultimately, the bending from ~1° in the native fibril to >12° per 10 repeating units in the presence of L-PGDS (as directly estimated from fibrils on the cryo-EM images and in MD simulations) (Fig. 2 and S14c) and the disruption of the critical interactions like intermolecular salt bridges (Fig. S14d), which are important for structural stability, results into the final breakage of the fibrils.

Interestingly, the typical free energy of amyloid folding ΔG is estimated to be −23.7 kcal/mol/chain from the amyloidatlas designed by Sawaya et al.^41^. Since the free energy from frustration release is lower than the estimated ΔG of amyloid folding, the amyloid disaggregation by L-PGDS will not produce monomers of the fibril but fibril chunks of variable size. This is consistent with our previous observation that the products of L-PGDS disaggregation consist of varying but still a well-defined number of protofibrils^22^. L-PGDS primarily binds to the lateral surface of the fibrils, which is more accessible than the fibril termini. Furthermore, it does not disaggregate fibrils deeper than relatively large protofibrils, which might be less toxic than smaller aggregates or monomers^46^. Hence, allowing L-PGDS to release the trapped proteins in amyloid deposits essentially reduces the opacity of the cornea (Fig. 5 and S20). This further elevates the therapeutic potential of L-PGDS when compared with other types of amyloid disaggregases, such as Hsp70 complexes which mainly target the fibril ends to initiate their amyloid disaggregation activity^30^. Moreover, from our previous studies of utilizing L-PGDS-ferritin conjugates in AD mice models^47^ and concentration-dependent disaggregation activity of L-PGDS shown in the ThT assays, we conclude that a significant amount of L-PGDS remains bound to the products of disaggregation thus preventing their re-aggregation as no regrowth of the amyloids was observed (Fig. S17a and S20). The L-PGDS-ferritin conjugates remain bound to the amyloid deposits in the mouse brain even after 72 h post-injection^47^. Thus, this mechanism of disaggregation of L-PGDS appears to be distinctly different from the entropic pull amyloid disassembly by Hsp70 and Hsp104 chaperones as well as by the bottle brush effect of the long alkyl chain detergents^29–31^, since in these systems the fibrils do break without undergoing significant persistence lengths changes, i.e. without remodeling before disassembly.

The supplementation of an endogenous chaperone holds an interesting opportunity to develop an effective and less toxic treatment of TGFBIp-related CD as it is non-invasive, non-toxic, endogenous, and abundant in many different tissues. Moreover, the outcome can be easily monitored in patients through basic ocular examination. As probed in the current study, the amyloid disaggregation ability of L-PGDS can be fine-tuned by structure-driven mutagenesis, which may further enhance the use of L-PGDS as a treatment in the context of specific genotypes associated with CD. Interestingly, our previous studies showed that the small molecular binders can effectively inhibit the production of pathological peptides during the proteolytic clearance of TGFBIp mutants by increasing the thermodynamic stability of the mutant back to the wild-type level^48^. In addition to its direct disaggregation effect, L-PGDS can potentially deliver these re-stabilizing binders to the folded TGFBIp protein in the stromal layer of the cornea, further enhancing the range of its therapeutic applications. L-PGDS has also been shown to disaggregate a variety of amyloid fibrils such as Aβ^22^, TGFBIp-peptide-derived fibrils from different regions (Fig. S18 and S21) and R3 region of Tau protein (Fig. S22). In the context of disease-causing amyloid fibril such as Aβ and tau, recent studies have utilized the extracted amyloid deposits from patients’ brain to serve as seeds to form the amyloid fibril for structural analysis^49–51^. Structural studies using the corneal amyloid obtained from patients as seeds for fibril formation and fibrils formed by different variants will be a focal subject of the future research and would enhance our understanding of the pathogenesis of the TGFBI-related CD.

In conclusion, our finding shows that the release of frustration energy stored in both the disaggregase and pathological amyloid fibrils could represent a novel source of energy used to break down amyloid fibrils via local restructuring and bending of the fibril. The proposed disaggregation mechanism of L-PGDS is evidently different from the entropic pulling and bottlebrush effect mechanism suggested for other disaggregation mechanisms. Our findings also present L-PGDS as a potential therapeutic strategy against amyloid-related disorders such as TGFBI-related CD.

## Materials and methods

### Overexpression and purification of labeled peptides

The preparation of ^15^N and ^13^C labeled G623R and A620D peptides was done as previously reported^33^. The protocol involves a His-tag-BEAK-tag-TEV-TGFBIp peptide construct (Biobasic Asia Pacific Pte Ltd, Singapore) where TEV cleavage site was inserted between the BEAK-tag and the desired peptide to facilitate the separation of the desired peptide from the tag. The synthesized plasmid was then transformed into *E. coli* BL21 (DE3) cells. The His-tag fusion protein was purified using Ni-NTA resin via immoblised metal affinity chromatography. After cleavage with TEV protease, the reaction mixture was passed through the Ni-NTA resin again to remove the tag. The flow through containing crude peptide was further purified by reverse phase-HPLC using C8 column to obtain high purity G623R peptides.

### Double-labeled G623R fibrils preparation

10 mg of ^15^N and ^13^C labeled G623R peptides was dissolved in 20 mM TRIS buffer containing 5% (w/v) G623R seeds. The seeds were obtained via sonication of the 1st generation unlabeled synthetic G623R fibrils. The solution with the 1st generation seeds were incubated for 5 days at 37 °C at 185 rpm to obtain homogenous fibrils sample for ssNMR packing.

For the G623R fibrils and L-PGDS complex sample, unlabeled L-PGDS was added to the pre-formed double labeled fibrils at a molar ratio of 1 :10 (1 mg of L-PGDS:10 mg of fibrils).

### ssNMR sample packing

The solution containing the double-labeled G623R fibrils was transferred to a rotor-packing device (Giotto Biotech). The packing device was then subjected to ultra-centrifugation at 100,000 × *g* for 4 h to pack the fibrils into a 1.9 mm zirconia rotor.

### ssNMR spectroscopy

3D NCACX, NCOCX and CANCO and 2D ^13^C-^13^C DARR (Dipolar Assisted Rotational Resonance), NCA and NCO experiments of the G623R fibrils were performed on an 18.8 T Bruker Advance III instrument equipped with a 1.9 mm HCN MAS probe (Table S6). These experiments were setup with reference to paper by Shi et al.^52^. The MAS spinning frequency was 17.857 kHz and variable temperature was set such that the actual temperature was 12 °C. Chemical shifts were referenced using the DSS scale with adamantane as a secondary standard for ^13^C (downfield signal at 40.48 ppm)^53^ and were indirectly calculated for ^15^N. ssNMR data were processed in Topspin 3.5 (Bruker Corporation) and then analyzed using the Sparky software (T. D. Goddard and D. G. Kneller, SPARKY 3, University of California, San Francisco). For the G623R fibrils and L-PGDS complex sample, 1D ^13^C CP (Cross-Polarisation) spectrum and 2D DARR (mixing time = 20 ms and 75 ms) were collected. The ssNMR data were then processed in Topspin 3.5 (Bruker Corporation) and then compared with the previously obtained spectrum of G623R fibrils using the Sparky software^54^. The equation used for the chemical shift perturbations is as described previously^37^.

### Double labeled A602D fibrils preparation and ssNMR studies

^13^C-^15^N-labeled peptide was expressed and purified. The fibrils were prepared from 10 mg of labeled sample. Fibrils were centrifuged and filled in 1.9 mm rotor to perform the experiments. ssNMR experiments for fibrils were performed on a 600 MHz Bruker Advance III HD spectrometer equipped with a 1.9 mm MAS HXY probe. The MAS spinning frequency was 13333 Hz. The variable temperature was regulated at 5 °C, which maintained the actual sample temperature at 12 °C. Chemical shifts were referenced using the DSS scale with admantane as a secondary standard for ^13^C. The typical 90° pulse lengths of ^1^H and ^13^C were 1.9 μs and 2.75 μs, respectively. ssNMR data were processed using Topspin 3.5.

### Monomer structure calculation

The experimentally assigned chemical shifts of the double-labelled G623R fibrils were used in TALOS+ software^55^ for secondary structure and backbone torsion angles prediction. The resulting ψ and φ angles from the TALOS+ prediction and the distance restraints information from the 2D ^13^C-^13^C DARR experiment were then used in the structural calculation of the monomer structure of the G623R fibrils using CYANA^35^. Out of the 20 structures generated, the monomer structure with the lowest energy was used for structure calculation for the G623R fibrils.

### Atomic force microscopy

Twenty microliters of G623R fibrils were deposited onto freshly cleaved mica and air-dried overnight. AFM images were obtained on Asylum Cypher S AFM (Oxfordshire, UK) in tapping mode using Nanoworld NCSTR silicon nitride soft-tip cantilevers (*R*_f_ = 160 kHz, *k* = 7.4 N m^−1^). The helical parameters of the fibril was then calculated from the AFM image.

### Cryo-electron microscopy

Quantifoil R1.2/R1.3 holey gold grids (Quantifoil) were glow-discharged for 1 min and plunge freeze using FEI Vitrobot Mark IV (Thermo Fisher Scientific). 4uL of fibril sample was applied on the glow-discharged grids for 10 s, followed by blotting for 3–4 s before plunging into the liquid ethane. Final high-resolution images were collected at Titan Krios—300 kV Cryo TEM equipped with Gatan K2 camera (Gatan). 40 movie frames were recorded with an exposure time of 4 s using a dose rate of 8 e/pixel/s at a pixel size of 0.85 Å on the specimen.

### Helical reconstruction

The movie frames were motion corrected and the Contrast Transfer Function was estimated using RELION-3.1 software^56^. Fibrils were manually picked, and segments were extracted with an interbox distance of ~9.5% of box size. A featureless cylinder was used as an initial model for 3D classification with an initial T value of 20. The T value was gradually increased to obtain the map for 3D refinement. Fourier shell correlation curves were computed between two half maps and according to the 0.143 criterion, the resolution of the final map is 4.9 Å.

### Model building

The single-chain atomic model of the G623R fibrils was obtained from the CYANA software using the distance and torsion angle restraints calculated from the ssNMR chemical shifts. Five copies of the single-chain atomic models were then fitted onto the EM density map using the Fit in Map function in Chimera^57^. The model was further refined using real-space refinement and CryoFit modules installed in the PHENIX software^58^. CryoFit is an automated cryo-EM density map fitting tool which utilizes MD simulation to increase the correlation between the density map and the initial atomic model. The CryoFit consists of eight-step procedures which involves energy minimization of the model, neutralizing the system and making restraints. The final atomic model of the G623R fibrils with the highest correlation was then obtained after the run.

### Synthetic peptides

Synthetic peptides with the G623R mutation from the 4th FAS-1 domain (^611/1^EPVAEPDIMATNGVVHVITNVLQ^633^) were purchased from Synpeptide Co Ltd, Shanghai, China. Other variants of the TGFBIp peptides (R124C, R124H, M619K, N622K and N622H) and Tau R3 peptides were purchased from Biobasic Asia Pacific Pte Ltd, Singapore. The peptide was lyophilized and allocated as described previously^11^. Thioflavin T (ThT) was purchased from Sigma-Aldrich (Sigma-Aldrich Inc., MO).

### In vitro uniform amyloid fibril formation

The 23-amino acid long peptide TGFBIp G623R from the 4th FAS1 domain of TGFBIp with the substitution, G623R (EPVAEPDIMATNRVVHVITNVLQ) that is known to rapidly aggregate to form amyloid fibrils was used in this study. The peptide powder was dissolved (0.15 mg/ml) in PBS and allowed to form amyloid fibrils in a shaking incubator at 37 °C and 180 rpm with and without the addition of L-PGDS at different concentrations of 4.64 µM, 9.28 µM and 23.3 µM. The molar ratio of L-PGDS to peptide was 1:10, 1:5 and 1:2, respectively.

### Overexpression and purification of human WT L-PGDS

The human WT L-PGDS with the c-terminal hexa-histidine tag was prepared as described previously^22,59^. Briefly, the glycerol stock of Rosetta 2 DE3, *E. coli* cells (Novagen) with pNIC-CH vectors were prepared via transformation and the transformed cells were overexpressed in TB media, by inducing with 0.5 mM IPTG at O.D_600_ of 0.8. The crude L-PGDS was injected into the AKTA purifier Fast Performance Liquid Chromatography (FPLC) (GE Healthcare, USA), and further purified using Superdex 75 column in Phosphate buffered saline (PBS) buffer. Sodium dodecyl sulfate-polyacrylamide gel electrophoresis (SDS-PAGE) was run to check the purity of the different fractions obtained after the FPLC run.

Plasmid of the different mutants of the WT L-PGDS were purchased from Biobasic Asia Pacific Pte Ltd. For ^15^N labeled L-PGDS, the glycerol stock of Rosetta 2 DE3, *E. coli* cells (Novagen) with pNIC-CH vectors was grown in M9 media supplemented with 1 g/L of ^15^N NH_4_Cl by inducing with 0.5 mM IPTG at O.D_600_ of 0.6. The subsequent purification steps were done in the same manner as the unlabeled L-PGDS.

### Transmission electron microscopy to assess structural changes of amyloid fibrils

Morphological analysis of the preformed G623R amyloid fibrils before and after treatment with different concentrations of L-PGDS for 72 h was investigated by Transmission electron microscopy (TEM) with a FEI T12 transmission electron microscope equipped with a 4K CCD camera (FEI) at a magnification of 49000X and electron dose of 20–30 e^−^/Å^2^. 4 µl of amyloid fibril samples with and without L-PGDS treatment were applied onto the glow-discharged 400-mesh-size carbon grids for 1 min and stained with 2% (v/v) uranyl acetate for 30 s before viewing under the microscope.

### Analysis of the EM images

The end-to-end distance (*D*), contour length (*L*) and angles of the fibrils were measured using the ImageJ software^60^. The persistence length (*P*) of the fibrils was obtained using the MS end-to-end distance processing tool in FiberApp software^61^. The plot of end-to-end distance is fitted using the following equation:

1 $$\left({D}^{2}\right)=4{PL}[1-\frac{2P}{L}\left(1-{e}^{\left(-\frac{L}{2P}\right)}\right)]$$

### Nuclear magnetic resonance (NMR) titration

NMR experiments were carried out in AVANCE II 600 MHz NMR spectrometer equipped with 5 mm z-gradient TCI cryoprobe. 2D ^1^H-^15^N HSQC spectra of ^15^N labeled L-PGDS were recorded in the absence and presence of the G623R fibrils at a molar ratio of 1:4 (L-PGDS: fibrils) and each spectrum was referenced to an external 4, 4-dimethyl-4-silapentane-1-sulfonic acid (DSS) signal. The HSQC spectra obtained were overlapped with the previously assigned spectra for the transference of assignment^37^ and the examination of chemical shifts perturbations induced by the peptides. The resultant spectra were processed using the Topspin (Bruker Biospin, USA) and CARA software (www.nmr.ch)^62^. The equation used for the chemical shift perturbations is as follows^37^:

2 $$\varDelta \delta ={\{[\varDelta \delta {(H)}^{2}]+[0.25* \varDelta \delta {(15N)}^{2}]\}}^{(\tfrac{1}{2})}$$

### Thioflavin T (ThT) to monitor amyloid fibril formation

The inhibitory effect of L-PGDS on amyloid fibril formation of TGFBIp was assessed by ThT assays. Peptide samples were allowed to fibrillate with the chaperone L-PGDS added at three different concentrations. The tested concentration of L-PGDS was 4.64 µM, 9.28 µM and 23.3 µM. The chaperone L-PGDS alone was also allowed to fibrillate together under the same conditions and was used as a baseline value to be subtracted for analysis. Fibril growth (with or without L-PGDS) was monitored at several time points using ThT fluorescence. The fibril solution containing L-PGDS and fibril solution alone were added to different wells in a Greiner 96-well flat-bottom polystyrol microplate (Greiner, Frickenhausen Germany) where 30 uM of Thioflavin T (ThT) solubilized in PBS buffer at pH 7.2 was subsequently added. The reaction was followed for 24, 48, 72 h. To examine the disaggregation properties of L-PGDS, ThT experiments were also performed 24, 48, and 72 h after the samples were allowed to form amyloid fibrils. The samples were excited at 445 nm and the resulting emission fluorescence at 485 nm was measured. Experiments were carried out in triplicates with at least two independent experiments using a microplate reader (Tecan infinite M200 pro, Zanker Road, San Jose, USA). The fluorescent reading from L-PGDS alone was subtracted from each reading before making percentage inhibition calculations. Percentage inhibition for each concentration of L-PGDS treatment was calculated by subtracting the baseline fluorescent intensity of L-PGDS alone without the peptides from the observed fluorescent intensity of each treatment well. The fluorescent intensities per L-PGDS treatment were normalized against the untreated fluorescent intensity per time point and expressed as a percentage.

### Molecular dynamics simulation

MD simulations were done to test the stability of the atomic model of G623R fibrils (PDB code: 8HIA) (Supplementary data 4). The starting model for the simulation was built using the CHARMM27 all-atom forcefield and TIP3P water model^63,64^. The CHARMM27 all-atom force field was used as the parameter sets have been optimized for simulations of proteins. The model was then inserted into a cubic water box with a distance of 1 nm from the solute to the box edge and it contained 16827 protein atoms and 320694 water molecules and additional 40 sodium ions to neutralize the system and to achieve a salt concentration of 0.15 M. Following that, the system was subjected to the steepest descent energy minimization using the GROMACS 2021.5 package until a force convergence of 1000.0 kJ/mol/nm is achieved to remove any bad contacts^65^. Classical molecular dynamics simulation was then performed for 50 ns where the temperature of the system was maintained at 300 K and 1 atm using the V-rescale thermostat and the Berendsen barostat respectively^66^. Bonds containing hydrogen atoms were constrained using the LINCS algorithm^67^, to enable a time step of 2 fs. Particle Mesh Ewald^68^ was used with a cutoff of 1.28 nm for electrostatics, and a cutoff of 1.28 nm was used for van der Waals interactions. The 50 ns simulation is split into 10 ns of equilibration run and 40 ns of production run. All simulation data obtained were analyzed and the timescale of 50 ns. All simulations were repeated three times with different random initial velocity to show that the results are independent of the initial configuration. The trajectory frames were saved every 2 ps. Commands used for distance and angle calculation are distance, minidist and gangle.

### Circular dichroism assays to monitor secondary structure changes of peptide

Far UV-CD spectra of the G623R peptide aggregation in the presence of various concentrations of L-PGDS at 24, 48 and 72 h were measured. Dissolution of amyloid fibrils incubated with various concentrations of L-PGDS for 24, 48, and 72 h after 24 h of G623R peptide fibrillation process, in PBS buffer at pH 7.2, was examined using a Chirascan plus spectropolarimeter (Chirascan, Applied Photophysics, UK), using a 0.1 cm path length quartz cuvette. Spectra were recorded from 260 nm to 190 nm in 0.1 nm steps at a scan rate of 50 nm/min. The final spectrum was the average of three scans as per the manufacturer’s recommendation. Since L-PGDS also has a distinct secondary structure consisting of mixed alpha and beta sheets, the spectra obtained for each L-PGDS concentration alone at different time points was subtracted from the peptide and L-PGDS treatment group at different time points. The Mean Residual Weight (MRW) ellipticity ([θ]_mrw_) at wavelength λ was calculated using the equation:

3 $$[{{{{{\rm{\theta }}}}}}]{{{{{\rm{mrw}}}}}}={{{{{\rm{MRWx}}}}}}{{{{{\rm{\theta }}}}}}{{{{{\rm{\lambda }}}}}}/\,10{{{{{\rm{xlxc}}}}}}$$

where θ_λ_ is the observed ellipticity at a particular wavelength (degrees), l is the path length (cm), and c is the concentration (g/ml).

### Fluorescent microscopy

For both inhibition of G623R peptide amyloid fibril formation by L-PGDS and dissolving of preformed amyloid fibrils, the microscopy samples after 72 h of incubation were obtained via the incubation of the peptide solution from the previous fibrillation assays in a 1:1 ratio with the ThT dye in the dark for 30 min. Twenty microliters of the sample was placed onto slides with coverslips and visualized under a fluorescence microscope (AxioImager Z1, Carl Zeiss, Oberkochen, Germany). Images for the different concentrations of the L-PGDS samples were also captured as the negative control. Images were captured and representative image from each time point under the given conditions were used for the quantitation of fluorescence. ImageJ^60^ software was used to quantify the signal from the images and the values for each time point per concentration of L-PGDS treatment were normalized to the untreated sample at that particular time point.

### Live-cell imaging by IncuCyte

For real-time observation of the effect of L-PGDS on cultured human corneal fibroblasts, 3000 cells/well were seeded in 96-well plates and placed in an incubator at 37 °C for 24 h to proliferate. Cells were then incubated with varying concentrations of L-PGDS (0.1 mM, 1 mM, 10 mM, 100 mM, and 1000 mM) in triplicates, and images were captured with IncuCyte ZOOM System (Essen BioScience Inc., Research Instruments, Singapore). Frames were then captured at 4-h intervals from 4 separate regions/well using a 10× objective for 20 h to observe cell toxicity.

### Immunohistochemistry and proteomics analysis of corneal sample

Corneal tissue was collected from patient with lattice corneal dystrophy (TGFBI mutation R124C) who was undergoing corneal transplantation at the Singapore National Eye Centre. Written informed consent was obtained from patient prior to surgery. The study was approved by the Centralised Institutional Research Board, Singhealth, Singapore (R2019/2386), and was carried out in accordance with the tenets of the Declaration of Helsinki.

Corneal stromal tissue samples were dissected into equal halves and incubated in 10 µM of L-PGDS or a buffer control, respectively, for 24 h in a shaking incubator at 37 °C. After incubation, samples were fixed in 3.7% formaldehyde overnight and subjected to a sucrose gradient treatment before embedding in OCT tissue freezing medium (Surgipath, Leica Microsystems) and stored at −80 °C until sectioning. Serial sections of 10um were cut using a HM525 NX cryostat (Thermo Scientific) onto Leica Bond Plus Slides (Leica Biosystems). TGFβIp amyloid deposits were stained with Congo red using an automated slide stainer according to the manufacturer’s protocol (Congo Red Staining Kit #860-026, Ventana Medical Systems, Inc) and visualized using an upright brightfield microscope (Nikon Eclipse Ti, Nikon).

### Sample preparation for mass spectrometry

The samples were added with Lysis buffer (EasyPep™ Mini MS Sample Prep Kit, Thermo Scientific, USA) to extract the proteins. The lysates were then centrifuged at 16,000 × *g* for 10 min at 4 ^o^C. The supernatant of the respective samples was aliquot into new tubes. Protein quantitation was done using DC Protein Assay (Bio-Rad Laboratories Inc., USA) for control sample. The protein solutions were then reduced, alkylated, digested and cleaned-up according to the manufacturer’s instruction (EasyPep™ Mini MS Sample Prep Kit, Thermo Scientific, USA).

Peptides were reconstituted in 2% Acetonitrile, 0.1% formic acid in water and peptide concentration was determined by Thermo Scientific™ Pierce™ Quantitative Fluorescent Peptide Assay.

### LC-MS/MS measurements

The reconstituted samples were then analyzed on an EASY-nLC 1200 system coupled to Orbitrap Exploris^TM^ 480 mass spectrometer (ThermoFisher Scientific, USA). The Easy-nLC system was equipped with an in-line trap column of PepMap 100 C18, 3 μm, 75 μm × 2 cm and Easy-spray Pepmap RSLC C18, 2 μm, 25 cm × 75 μm column. The EASY-nLC was operated at a flowrate of 300nL/min. Mobile phase A consisted of 0.1% formic acid in LC-MS grade water and mobile phase B was made up of 0.1%formic acid, 80% acetonitrile in LC-MS grade water. The gradient comprised of a 35 min step gradient from 5% to 18% mobile phase B, 20 min from 18% to 50% solvent B and lastly 5 min from 50% to 95% solvent B. Orbitrap Exploris^TM^ 480 mass spectrometer was operated in data-independent and positive ionization mode. MS measurements were performed as described below: MS1 spectra were recorded at a resolution of 120k with MaxIT mode set to auto. The *m/z* scan range was 350 to 1200 for full scan. The automatic gain control (AGC) target was set to custom with normalized AGC target at 300%. Peptides were then selected for tMS2 using HCD Collision energy at 30% with a fixed collision energy mode and the fragments were detected in the Orbitrap at a resolution of 30k with auto MaxIT. The isolation window was set to custom with m/z set to 45.7. The scan range mode was set to ‘Define First Mass’ at m/z 110. The AGC target was set to custom with normalized AGC target set to 1000%. The resulting MS/MS data were processed using Spectronaut (Biognosys AG, Switzerland) DirectDIA analysis.

### Statistics and reproducibility

All of the data are presented as the mean ± standard error of the mean (SEM), with numbers of repeats indicated in the figure legends.

### Reporting summary

Further information on research design is available in the Nature Portfolio Reporting Summary linked to this article.

## Supplementary information

Supplementary Information

Description of Additional Supplementary Files

Supplementary Data 1

Supplementary Data 2

Supplementary Data 3

Supplementary Data 4

Supplementary Video

Reporting Summary

## Data Availability

The atomic coordinates and structure factors have been deposited in the RCSB Protein Data Bank (PDB), Biological Magnetic Resonance Bank (BMRB) and Electron Microscopy Data Bank (EMDB). The PDB codes of the G623R fibril and its repeating unit are *8HIA and 8HGA, respectively. The EMDB code of the G623R fibril is EMD-34813 and the BMRB ID of the G623R fibril repeating unit is 36518. Proteomics data are available via ProteomeXchange with identifier PXD038281. Source data for Fig. 4b can be found in Supplementary Data 1. All other data or materials can be obtained from the corresponding author upon request.
