# Supplementary material for: Release of frustration drives corneal amyloid disaggregation by brain chaperone
Source: Commun Biol. 2023 Mar 30;6:348. doi: 10.1038/s42003-023-04725-1 (PMC10063603; doi:10.1038/s42003-023-04725-1)
Supplement: Supplementary file 1 — Supplementary Information [file 42003_2023_4725_MOESM1_ESM.pdf]

Supplementary Figures

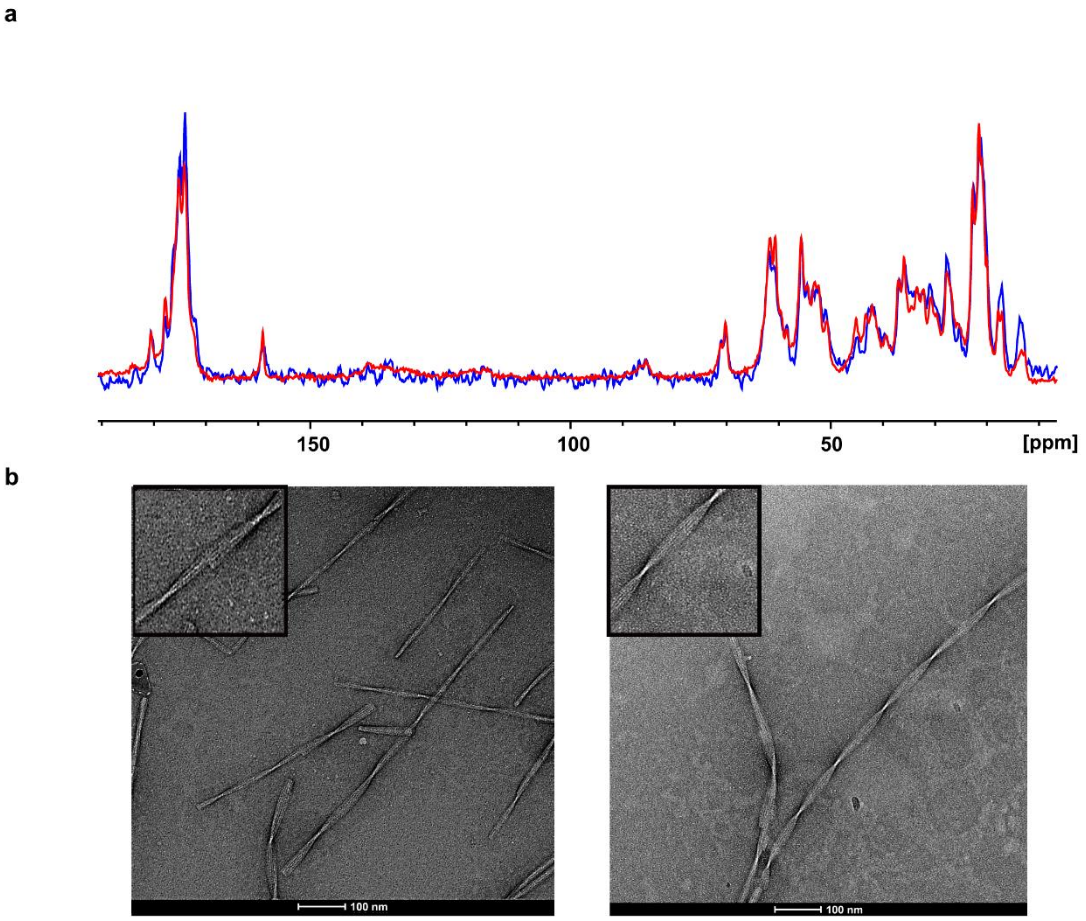

Figure S1: 1D CP spectra **a** and **b** negative stained images of the 1st (blue) and 2nd generation (red) of G623R fibrils respectively.

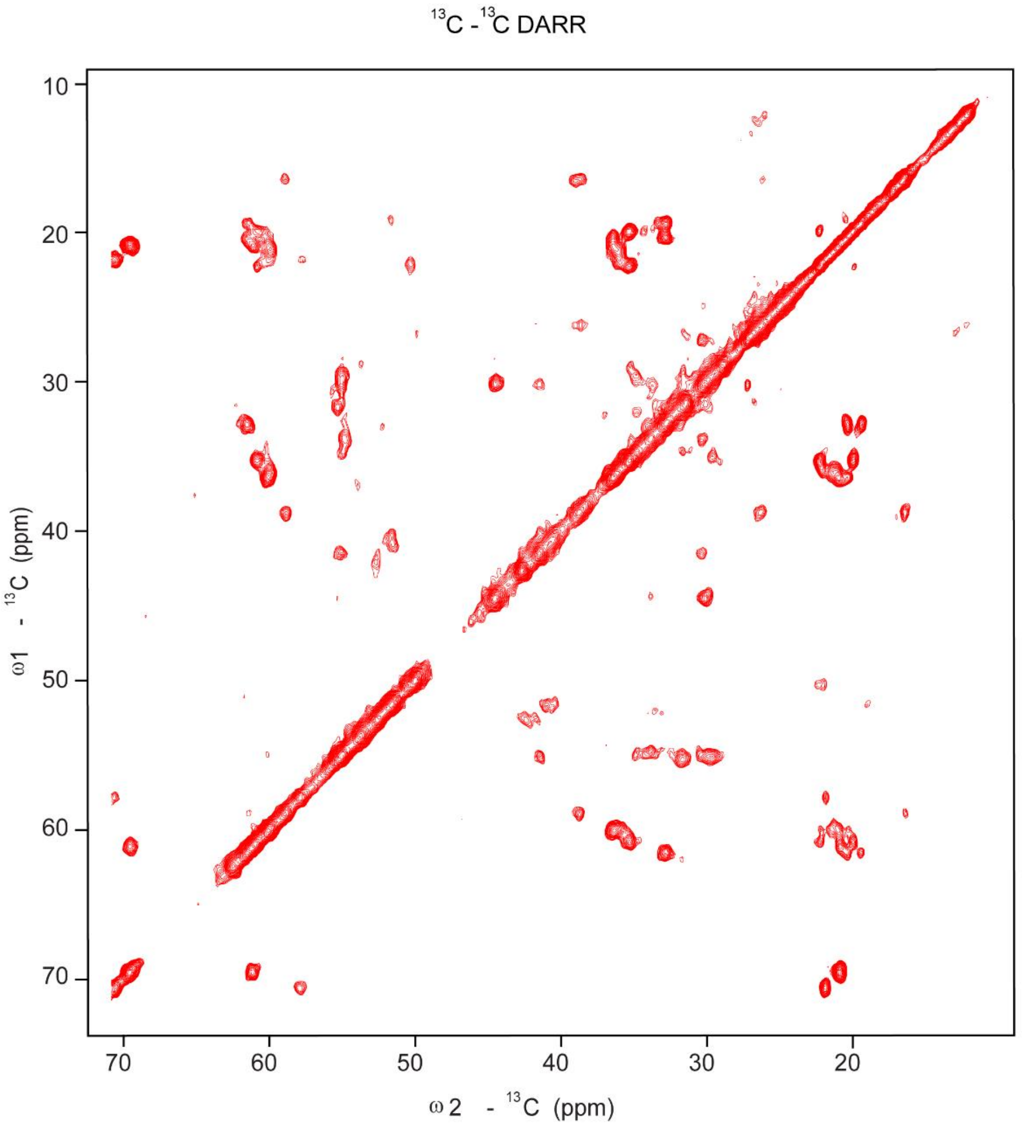

Figure S2: Selected regions of of 2D ^{13}C-^{13}C DARR spectrum of ^{13}C-^{15}N labeled G623R fibrils with 20 ms contact/mixing time. (Contour level: 1.5e+006, 37, 1.1).

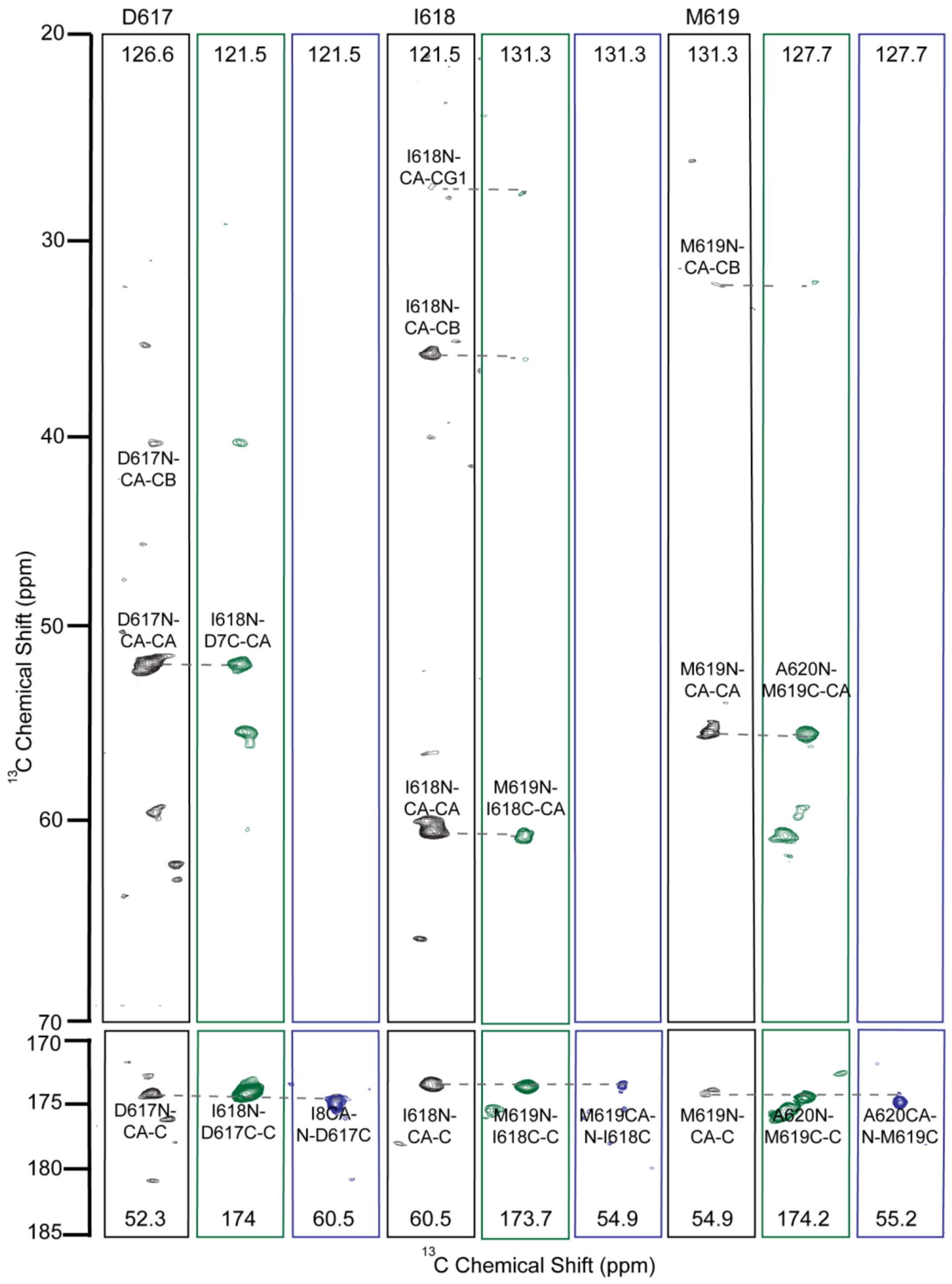

D617
I618
M619
126.6
121.5
121.5
121.5
131.3
131.3
131.3
127.7
127.7
I618N-CA-CG1
M619N-CA-CB
I618N-CA-CB
D617N-CA-CB
D617N-CA-CA
I618N-D7C-CA
M619N-CA-CA
A620N-M619C-CA
I618N-CA-CA
M619N-I618C-CA
D617N-CA-C
I618N-D617C-C
I8CA-N-D617C
I618N-CA-C
M619N-I618C-C
M619CA-N-I618C
M619N-CA-C
A620N-M619C-C
A620CA-N-M619C
52.3
174
60.5
60.5
173.7
54.9
54.9
174.2
55.2
^{13}C Chemical Shift (ppm)
^{13}C Chemical Shift (ppm)

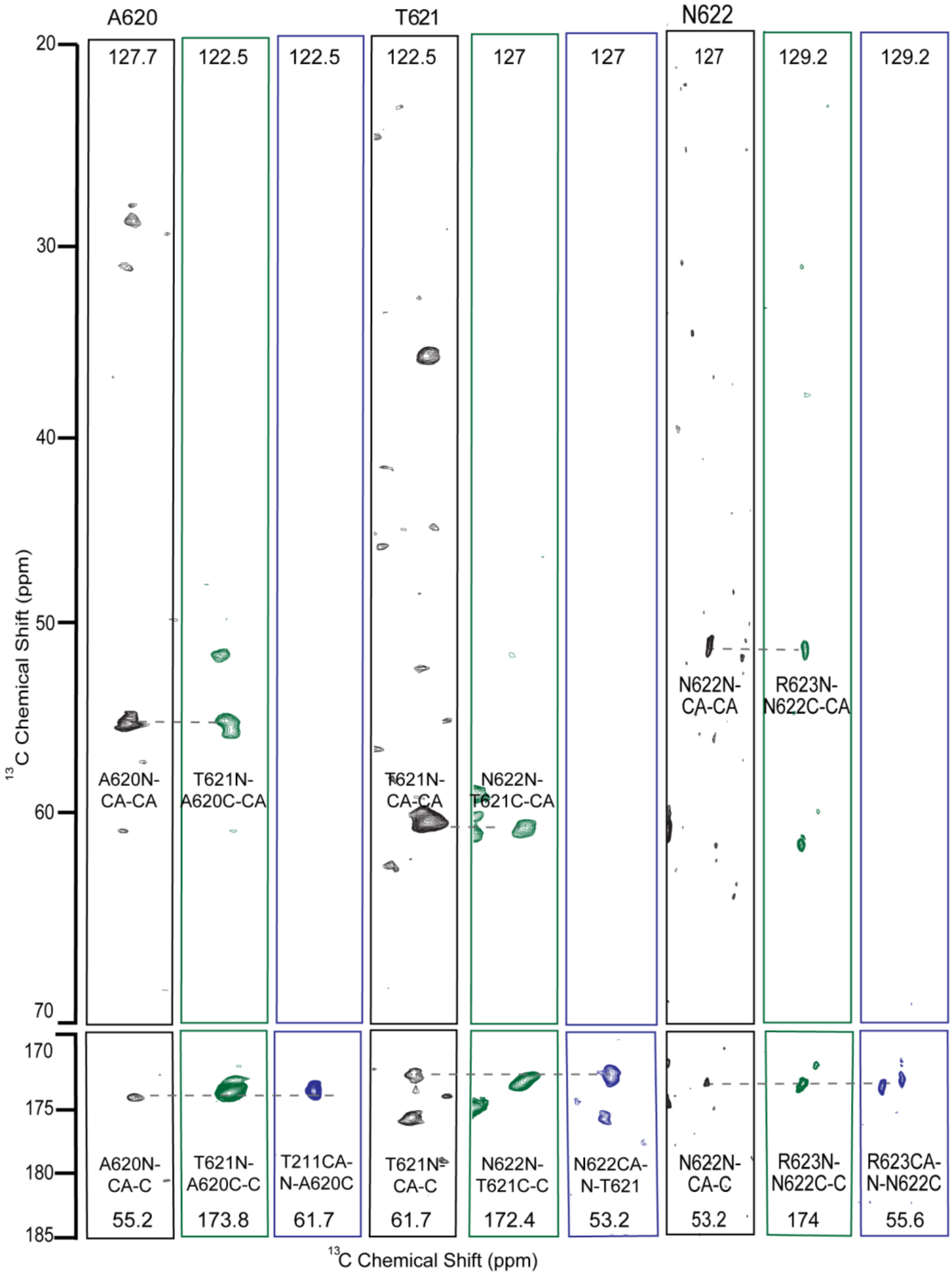
A620
T621
N622
127.7
122.5
122.5
122.5
127
127
127
129.2
129.2
A620N-CA-CA
T621N-A620C-CA
T621N-CA-CA
N622N-T621C-CA
N622N-CA-CA
R623N-N622C-CA
A620N-CA-C
T621N-A620C-C
T211CA-N-A620C
T621N-CA-C
N622N-T621C-C
N622CA-N-T621
N622N-CA-C
R623N-N622C-C
R623CA-N-N622C
55.2
173.8
61.7
61.7
172.4
53.2
53.2
174
55.6
20
30
40
50
60
70
170
175
180
185
13C Chemical Shift (ppm)
13C Chemical Shift (ppm)

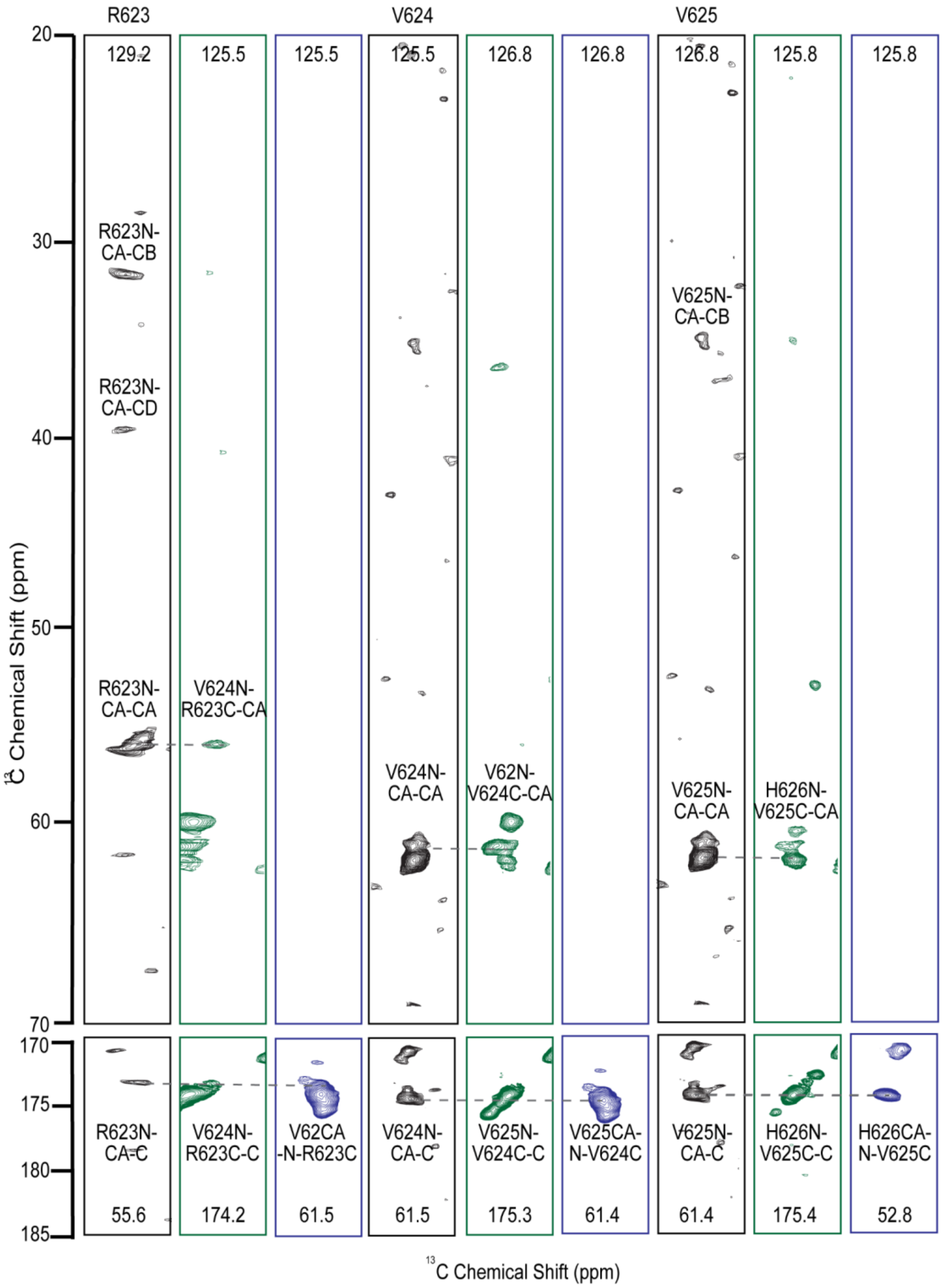
R623
V624
V625
129.2
125.5
125.5
125.5
126.8
126.8
126.8
125.8
125.8
R623N-CA-CB
R623N-CA-CD
R623N-CA-CA
V624N-R623C-CA
V624N-CA-CA
V62N-V624C-CA
V625N-CA-CB
V625N-CA-CA
H626N-V625C-CA
R623N-CA-C
V624N-R623C-C
V62CA-N-R623C
V624N-CA-C
V625N-V624C-C
V625CA-N-V624C
V625N-CA-C
H626N-V625C-C
H626CA-N-V625C
55.6
174.2
61.5
61.5
175.3
61.4
61.4
175.4
52.8
13C Chemical Shift (ppm)
13C Chemical Shift (ppm)

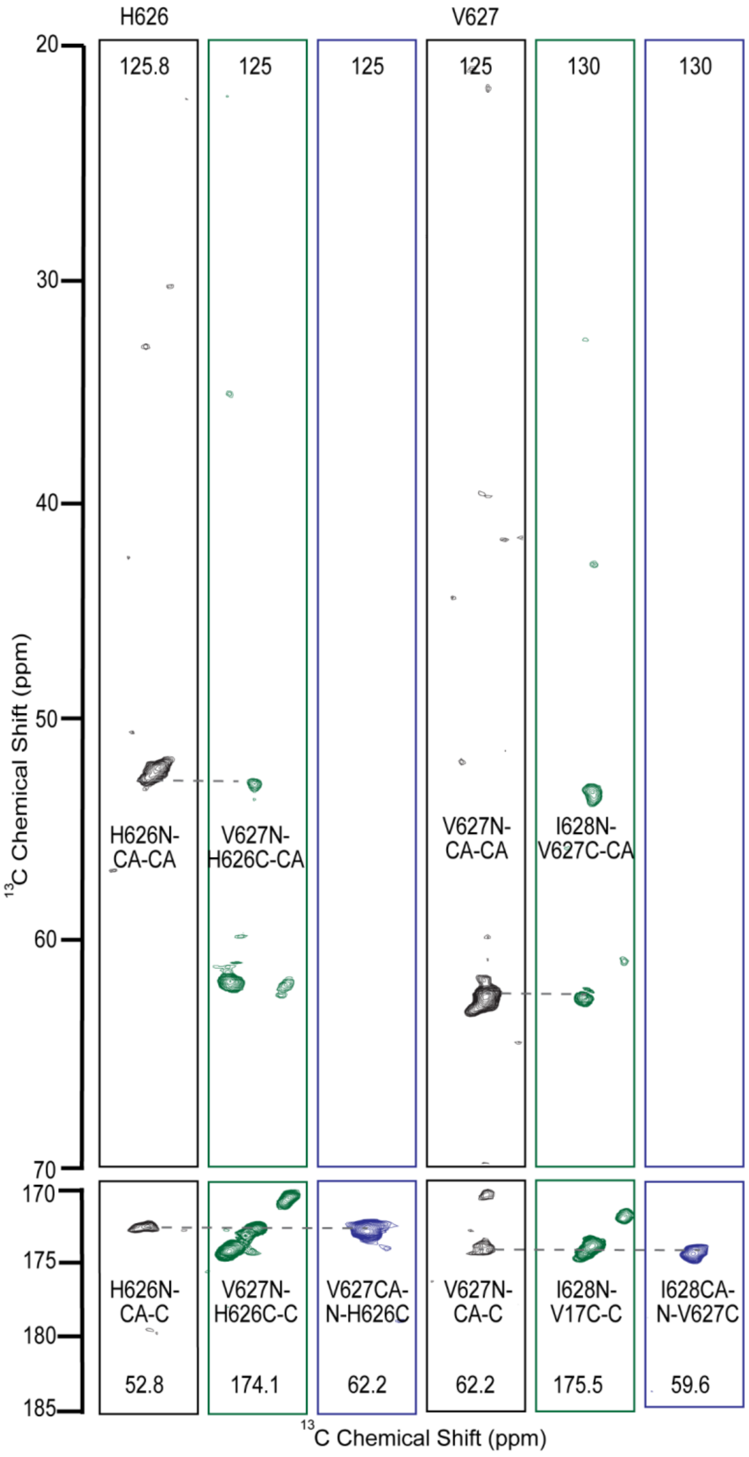

Figure S3: Representative sequential assignment walk for the G623R fibril. Strip plots of 3D NCACX (black), NCOCX (Green) and CANCO (blue) spectra used for sequential assignment for the residues D7 –V17. The sequential lines are shown as grey dashed lines.

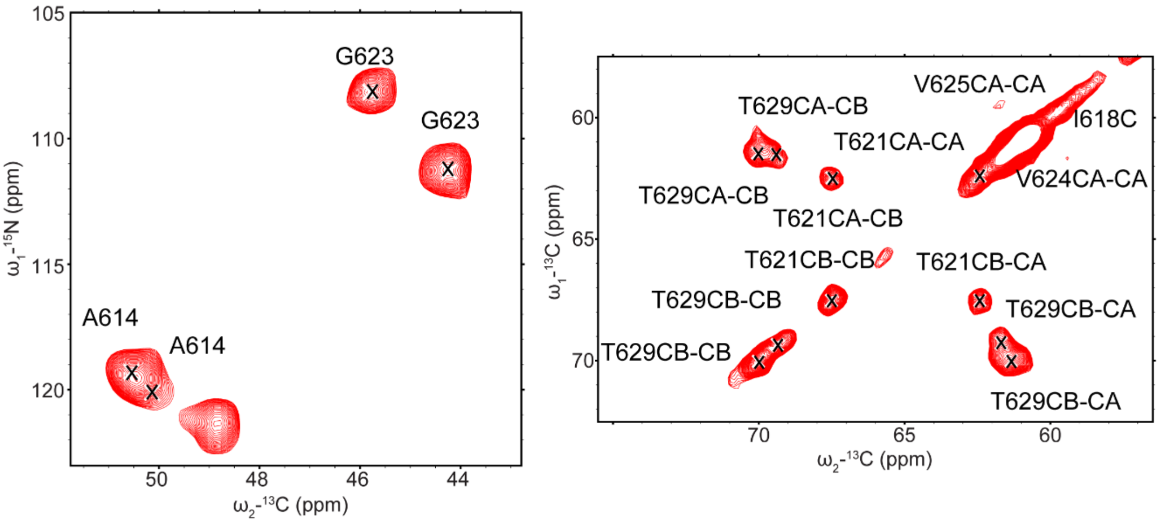

Figure S4: Selected region of 2D ^{13}C-^{13}C NCA displaying resonance doubling and 2D ^{13}C-^{13}C DARR spectrum of A620D fibrils with 20 ms ^{13}C-^{13}C DARR mixing time. (Contour level: 1.5e+006, 37, 1.1)

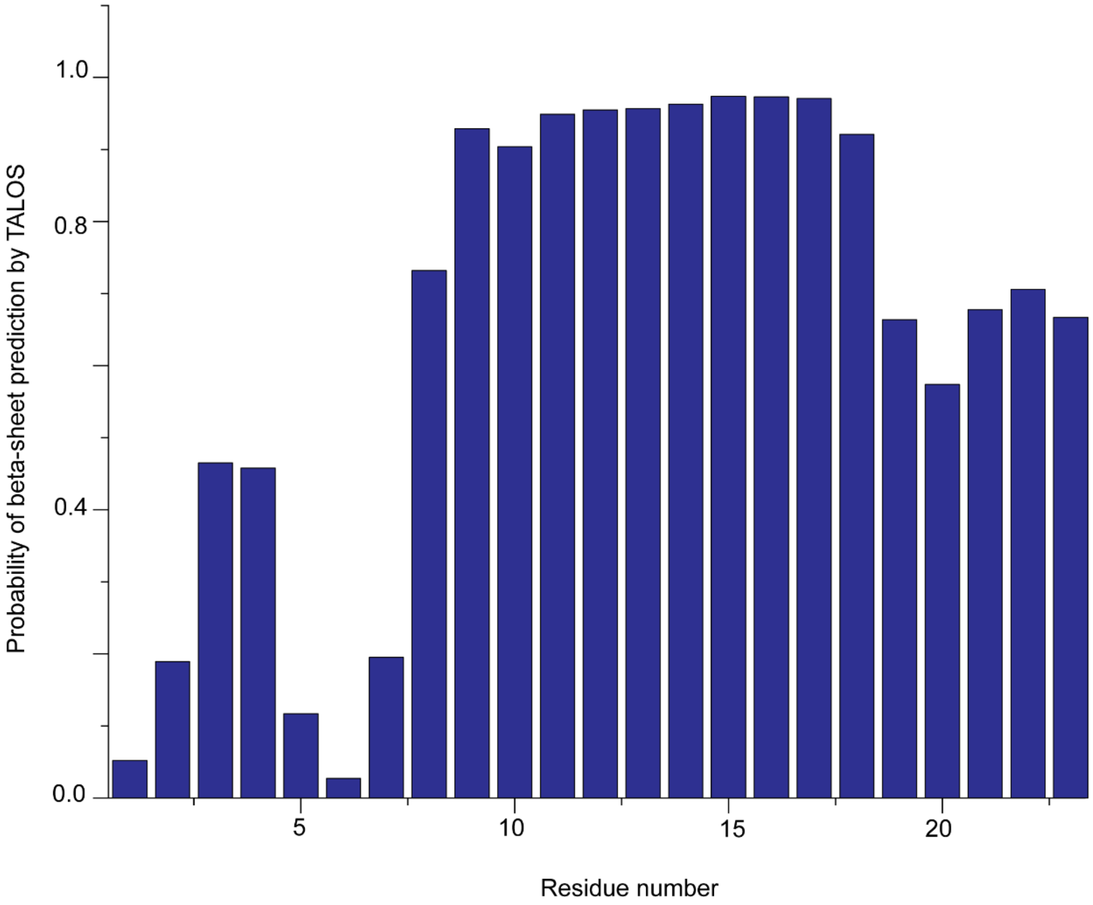

Figure S5: TALOS prediction of the probability of beta-sheet for each residue in G623R fibrils.

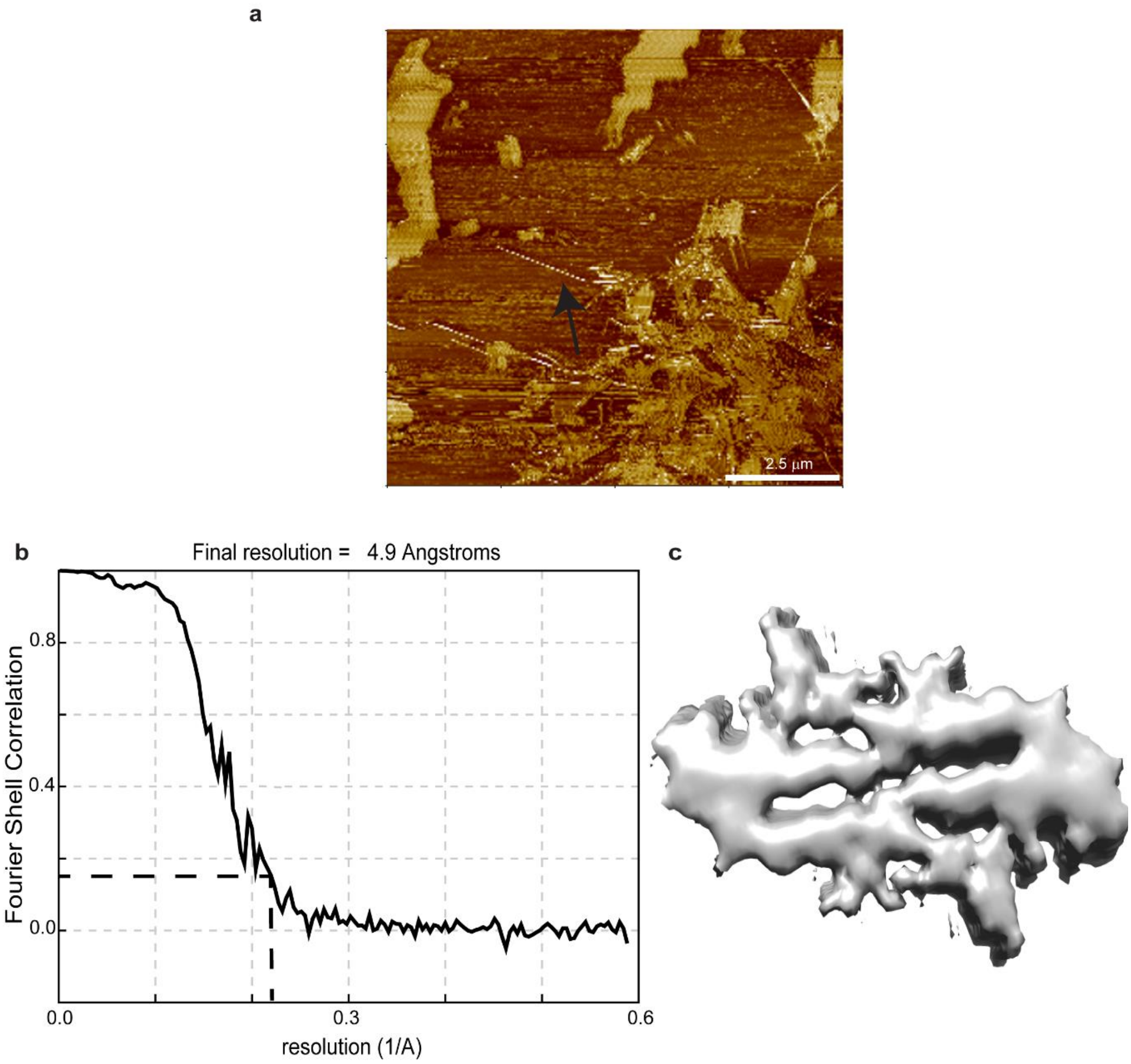

Figure S6: AFM and Cryo-EM data for G623R fibril. **a** The AFM images of the G623R fibril showing the left-handed twist of the fibril shown by the black arrow. **b** The Fourier Shell Correlation (FSC) curve for the electron density calculated without additional symmetry and with near 2_1 symmetry. Black dashed lines indicate resolution calculated to be 4.9 Å. **c** The electron density map of G623R fibril.

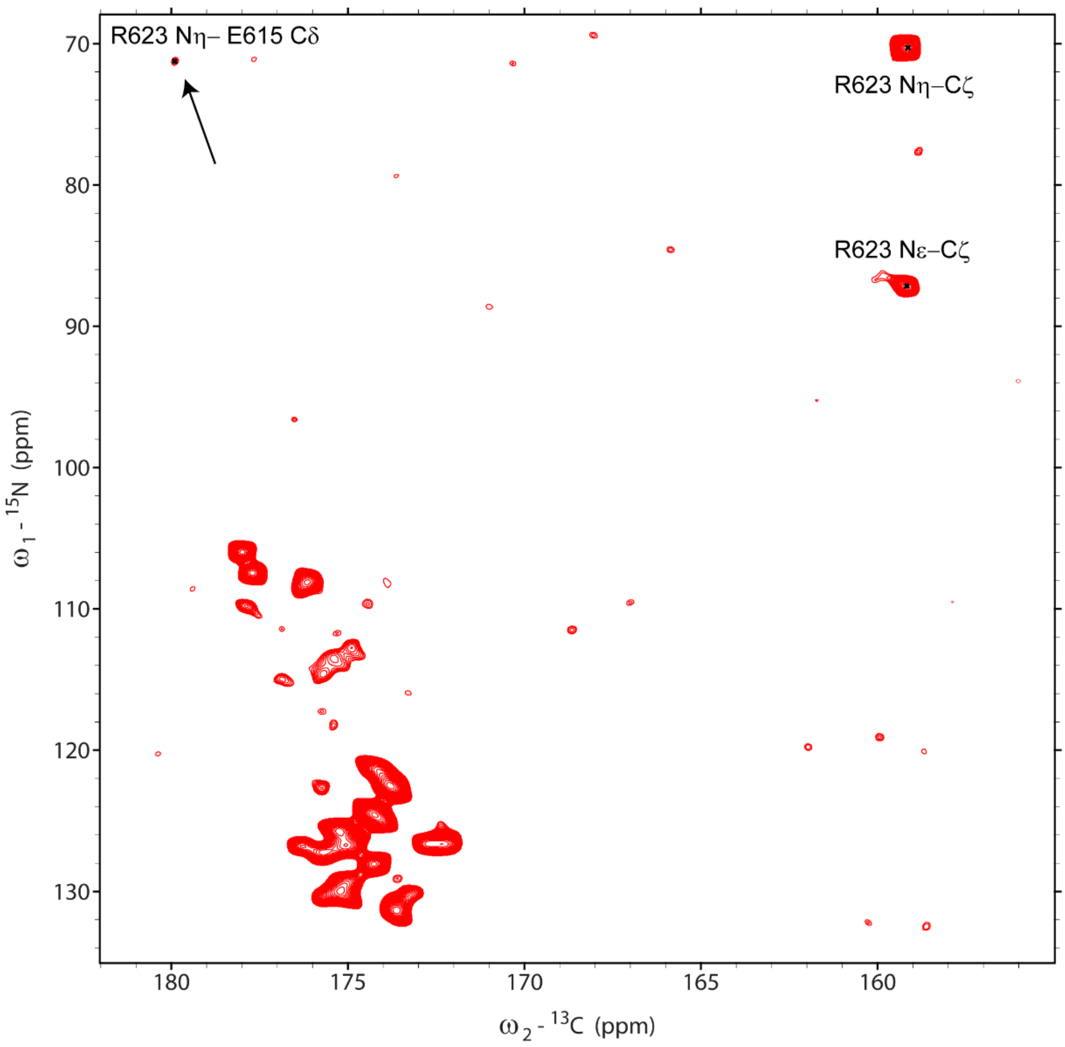

Figure S7: 2D NCO spectra with 20 ms mixing time showing the cross peak between E615 and R623. (Contour level: 1.9e+006, 37, 1.1)

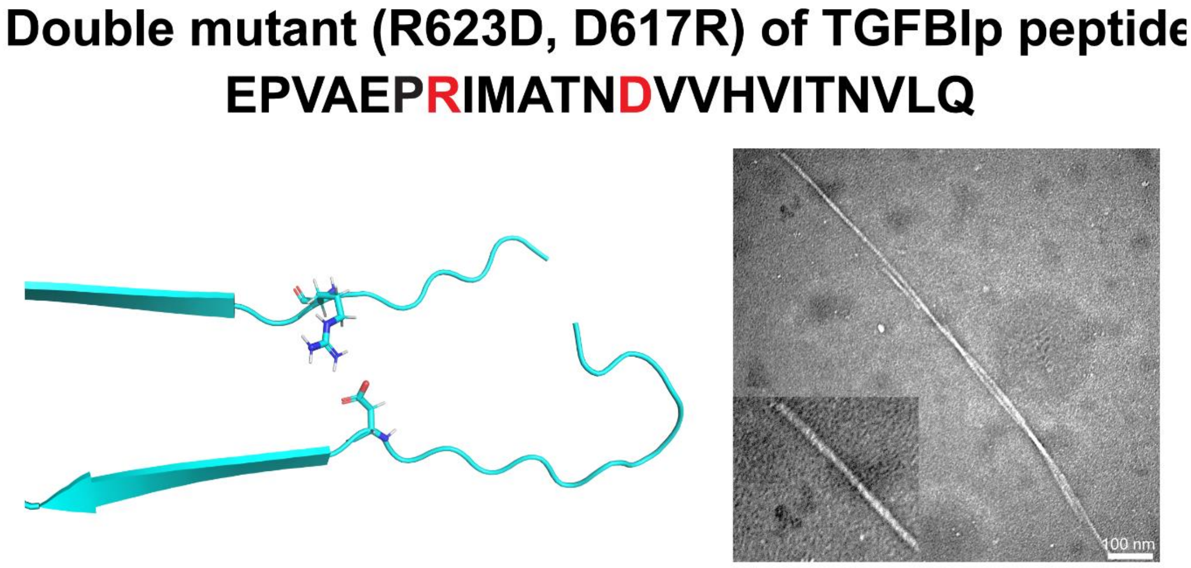

Figure S8: The peptide sequence of the double mutant (R623D, D617R) TGFBIp peptide, cartoon representation and the TEM image of the fibril formed by double mutant peptide.

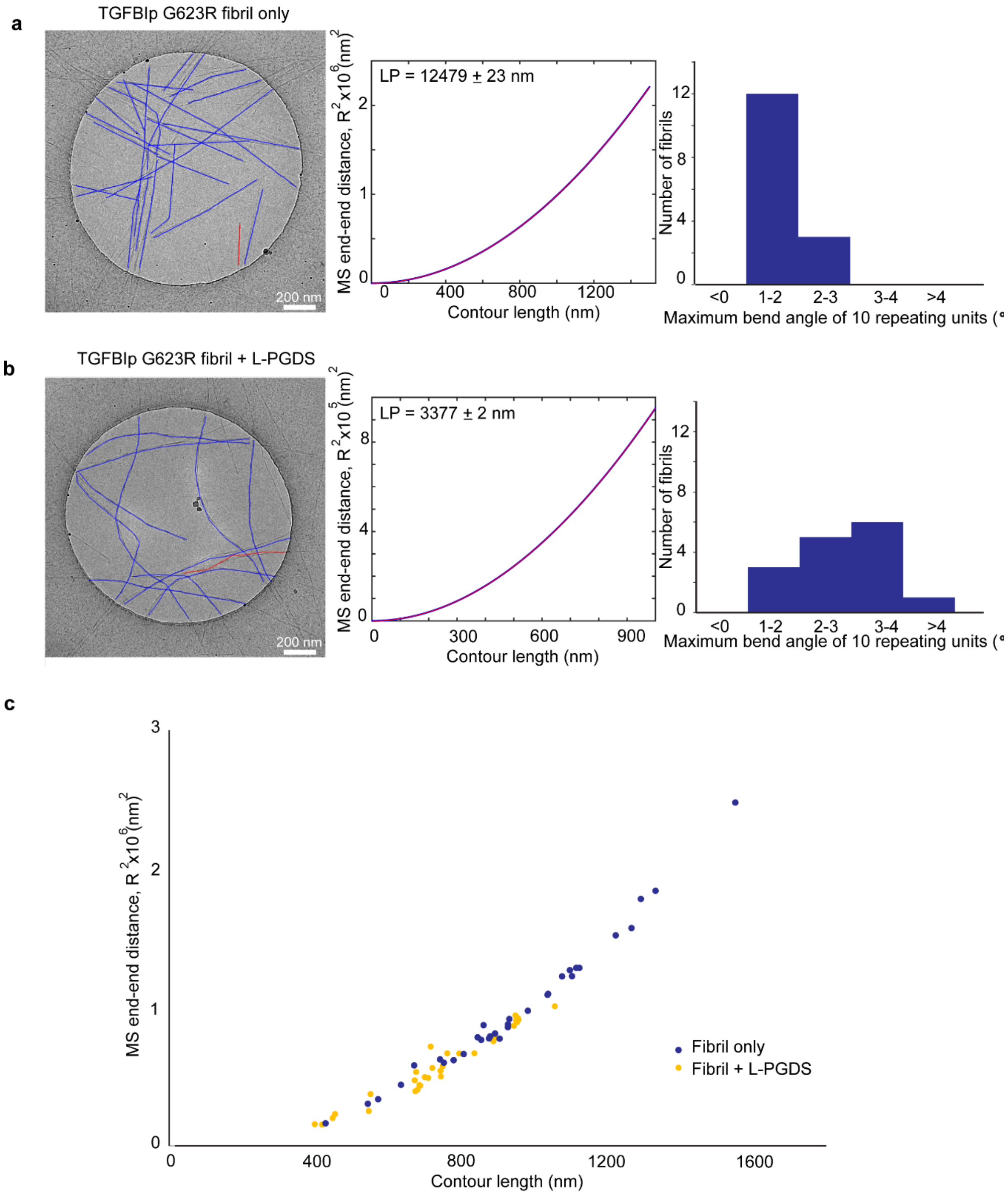

Figure S9: Persistence length and distribution of the maximum bend (curvature) angles of 10 repeating units in the cryo-EM field of view calculated using the FiberApp[1] for **a**, G623R fibrils and **b** G623R fibril + L-PGDS complex. **c** The linear plot of end to end distance, R^2 ,against contour length of the fibril in absence or presence of L-PGDS.

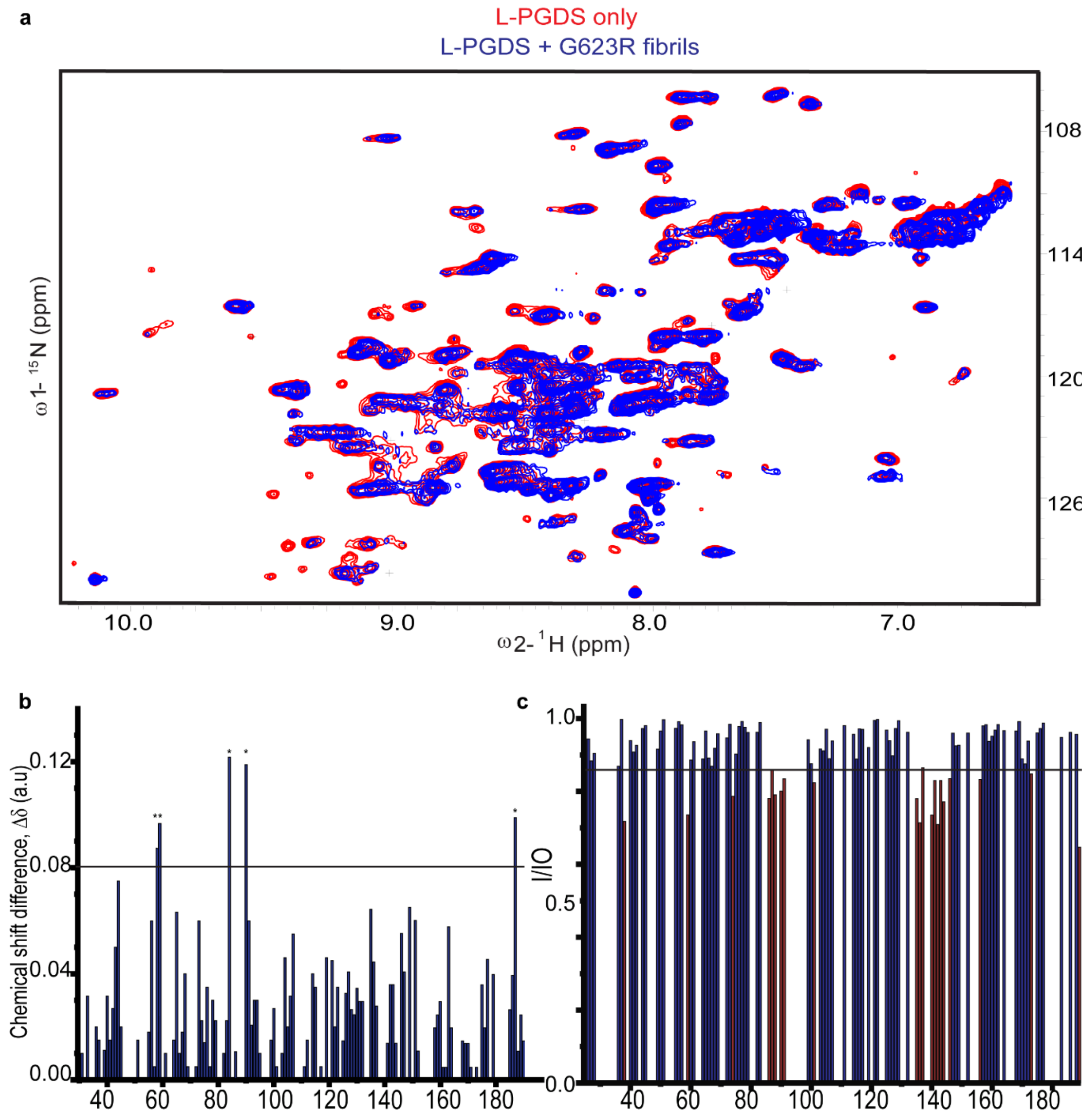

Figure S10: **Solution NMR spectra of the interaction between L-PGDS and G623R fibril.** **a** ^{1}H-^{15}N HSQC spectra of WT L-PGDS (red) and L-PGDS + G623R fibril (blue). **b** Graph showing chemical shift perturbation for ^{15}N L-PGDS in the presence of G623R fibrils. Residues that showed sigificant chemical shift perturbation are highlighted in asterisks (*). **c** Graph showing changes intensity ratio (I/IO) comparing L-PGDS alone and L-PGDS in the presence of G623R fibrils. Residues showing I/IO < 0.8 are highlighted in red. IO is the intensity of free L-PGDS and I is the intensity of L-PGDS and G623R complex.

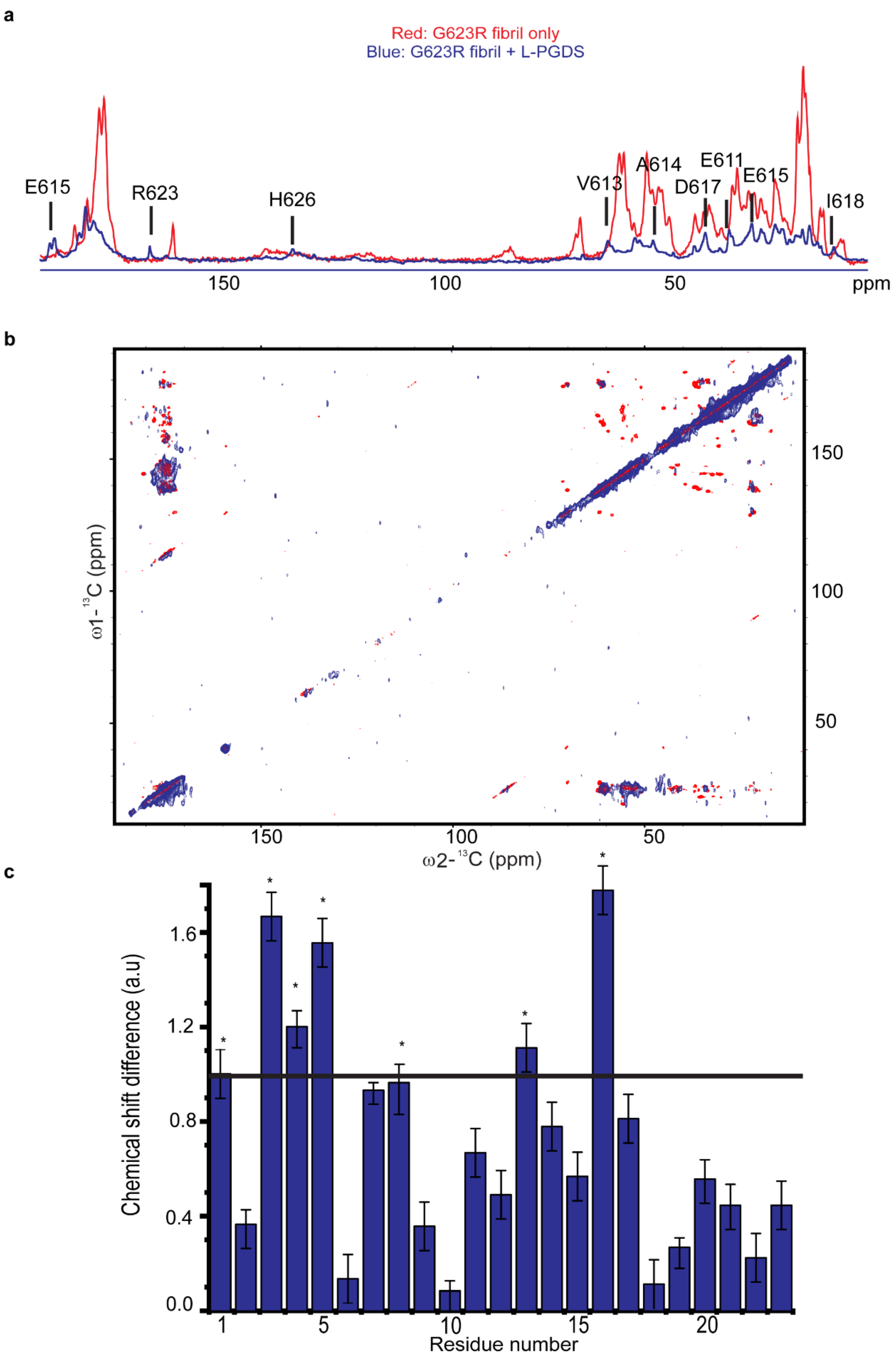

Figure S11: **ssNMR spectra of the interaction between G623R fibril and L-PGDS. a** 1D ^{13}C CP spectra **b** ^{13}C-^{13}C DARR spectra of G623R fibril (red) and G623R fibrils + L-PGDS (blue) with 20 ms ^{13}C-^{13}C DARR mixing time. (Contour level: 4e+006, 37, 1.1). **c** Graph showing chemical shift perturbation for ^{13}C-^{15}N G623R fibril in the presence of L-PGDS.

Residues that showed sigificant chemical shift perturbation are highlighted in asterisks (*). Error bars correspond to the chemical shift variation measured from three different readings from the same experiment and number of experiment (n) =1.

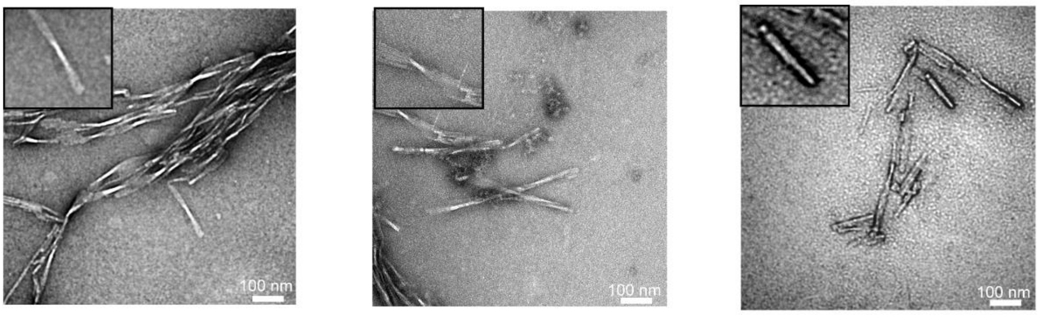

TGFBIp G623R Fibril

TGFBIp G623R Fibril and WT L-PGDS

TGFBIp G623R Fibril and RA L-PGDS

Figure S12: Representative negative stain EM images of G623R fibril control incubated in PBS buffer only, WT L-PGDS and RA L-PGDS respectively.

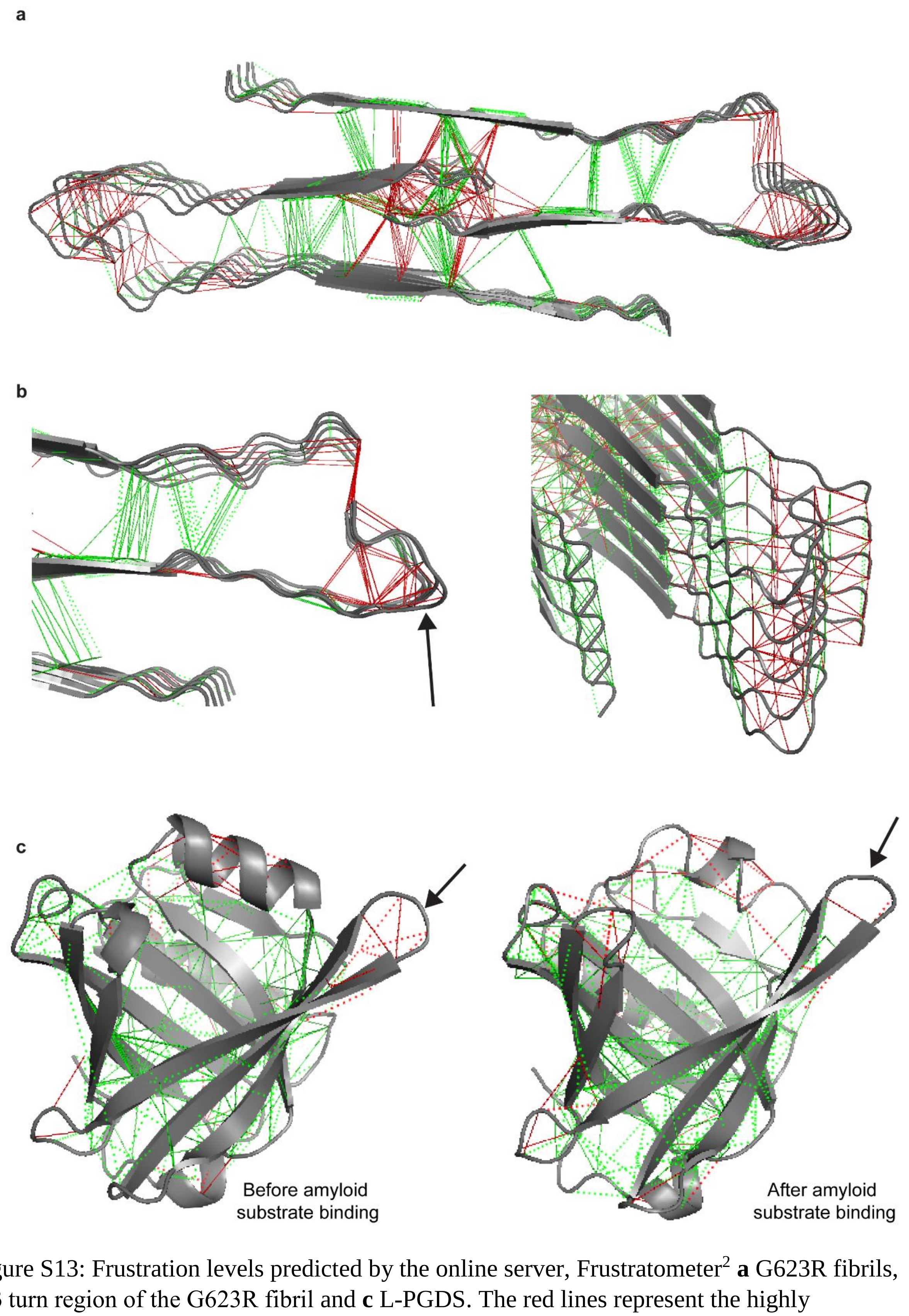

Figure S13: Frustration levels predicted by the online server, Frustratometer[2] **a** G623R fibrils, **b** β turn region of the G623R fibril and **c** L-PGDS. The red lines represent the highly frustrated regions (as shown by the black arrow), and the green lines represent the minimally frustrated regions.

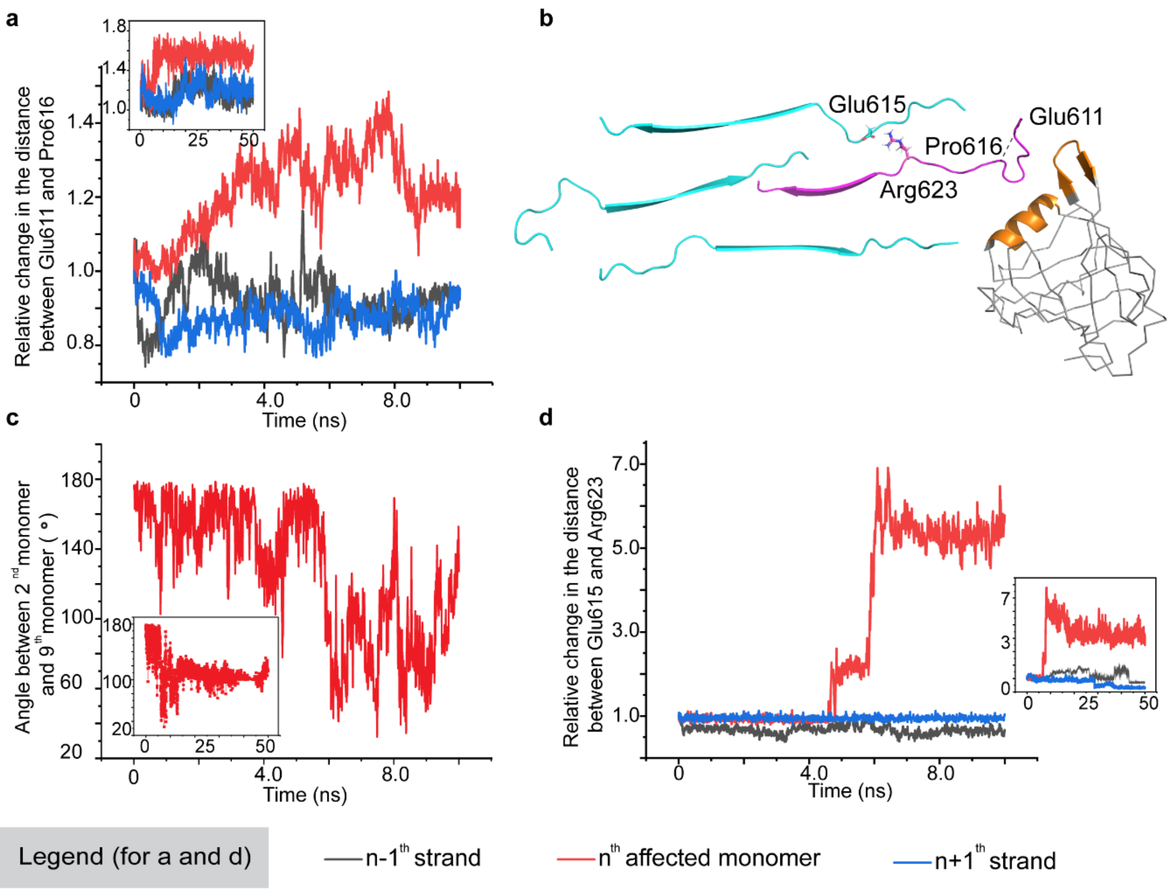

Figure S14: MD simulation of G623R fibril and L-PGDS. **a** Relative change in the distance between residue pairs Glu611-Pro616 to the Glu611-Pro616 distance at T=0, $n \geq 3$ independent experiments. **b** 3D representation of the β-turn flipping out event. The interacting helix and loop of L-PGDS are highlighted in orange and the affected monomer is highlighted in magenta. **c** The angle between the normal to the planes of 2nd and 9th monomer defined by CA of A10, N12 and H16 as shown in Fig. S19. **d** Relative change in the distance between averaged heavy-atom positions of E615 and R623 to the E615 and R623 distance at T=0. The insets in A, C and D shows the entire 50 ns MD trajectory.

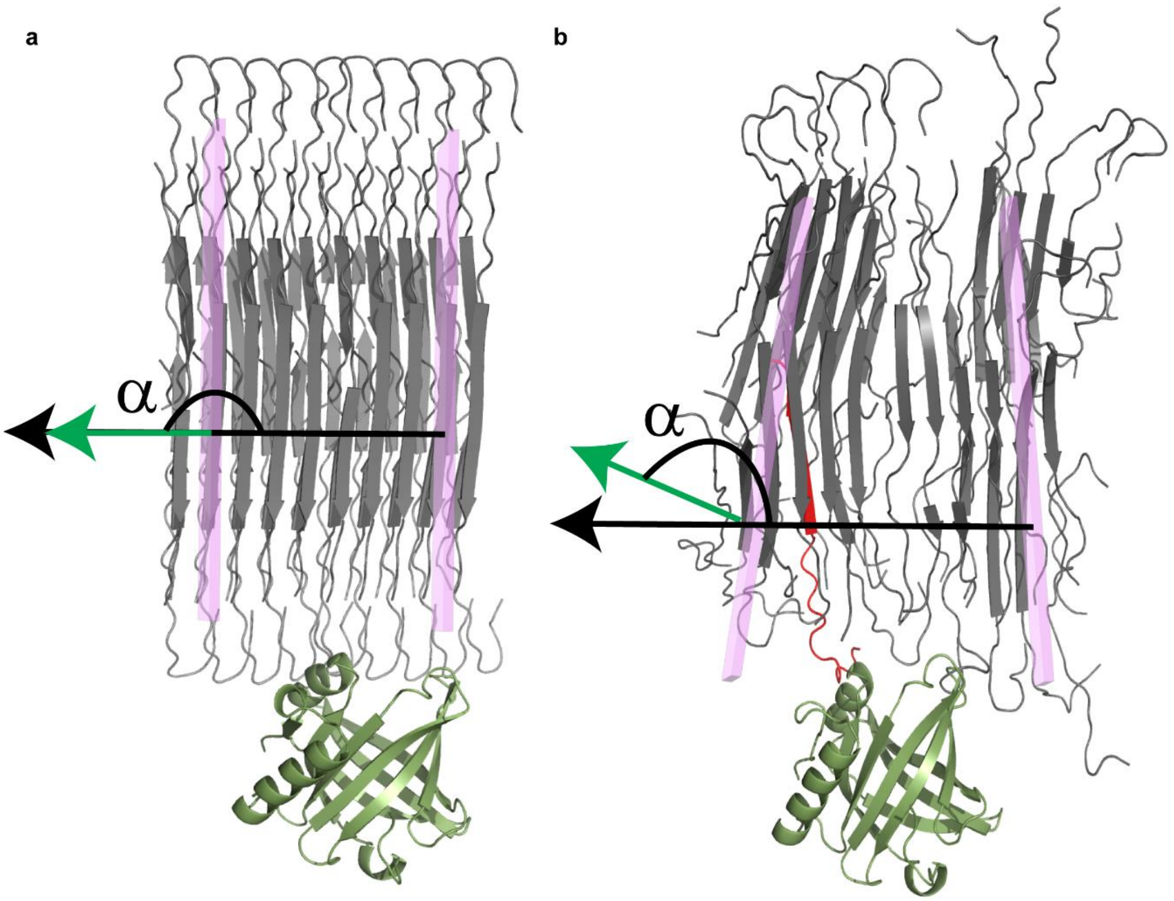

Figure S15: MD simulation snapshots taken at T=0 and T=6 ns. The angle, α, is defined between the normal to the planes of 2nd and 9th monomer defined by CA of A10, N12 and H16 (pink). The black and green arrows represent the normal of the plane of the 2nd and 9th monomer. The interacting monomer is highlighted in red.

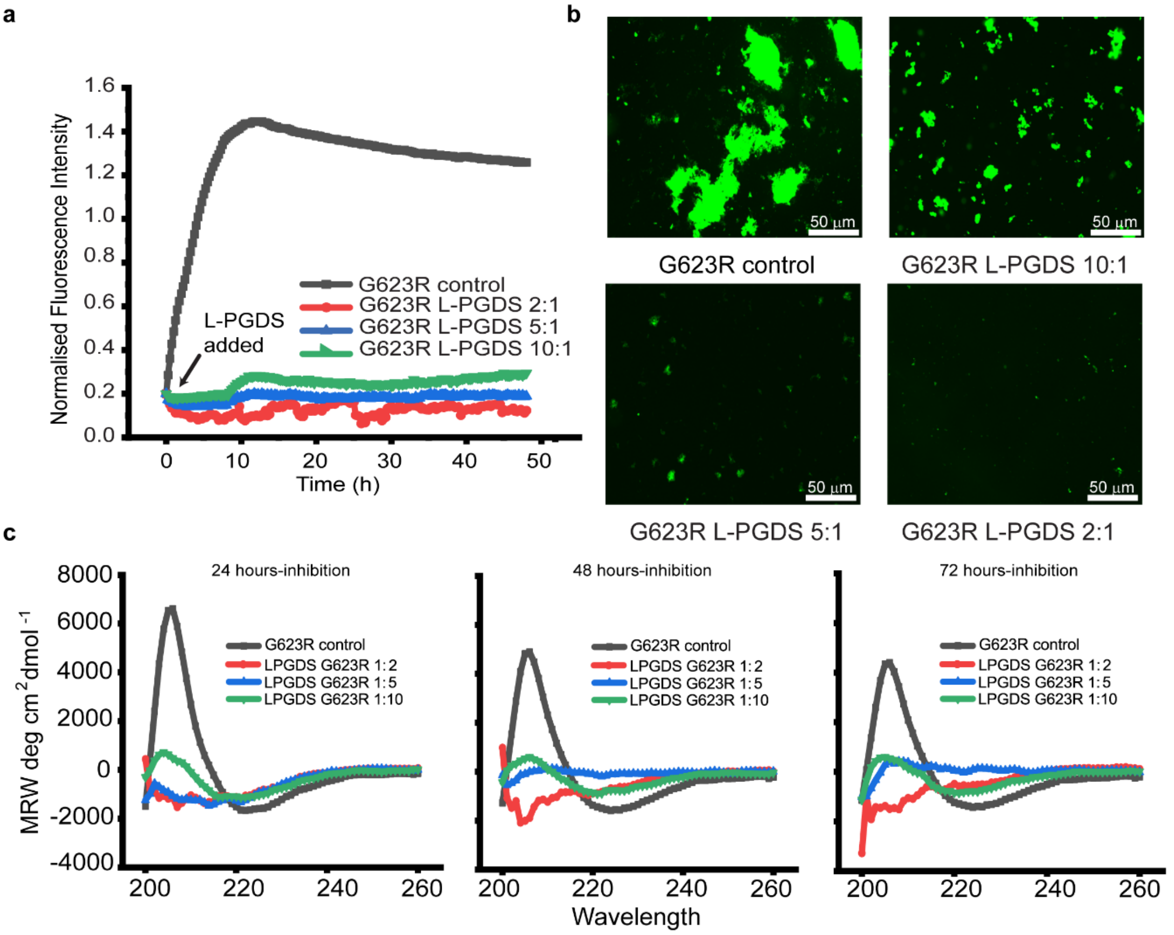

Figure S16: The inhibitory effects of L-PGDS on G623R peptides. **a** Long hour kinetic ThT assays. **b** Representative fluorescence microscopy images showing the inhibitory effects of L-PGDS on G623R peptides after 72h incubation. **c** Circular Dichroism spectra showing the aggregation inhibitory effects of L-PGDS on G623R peptides.

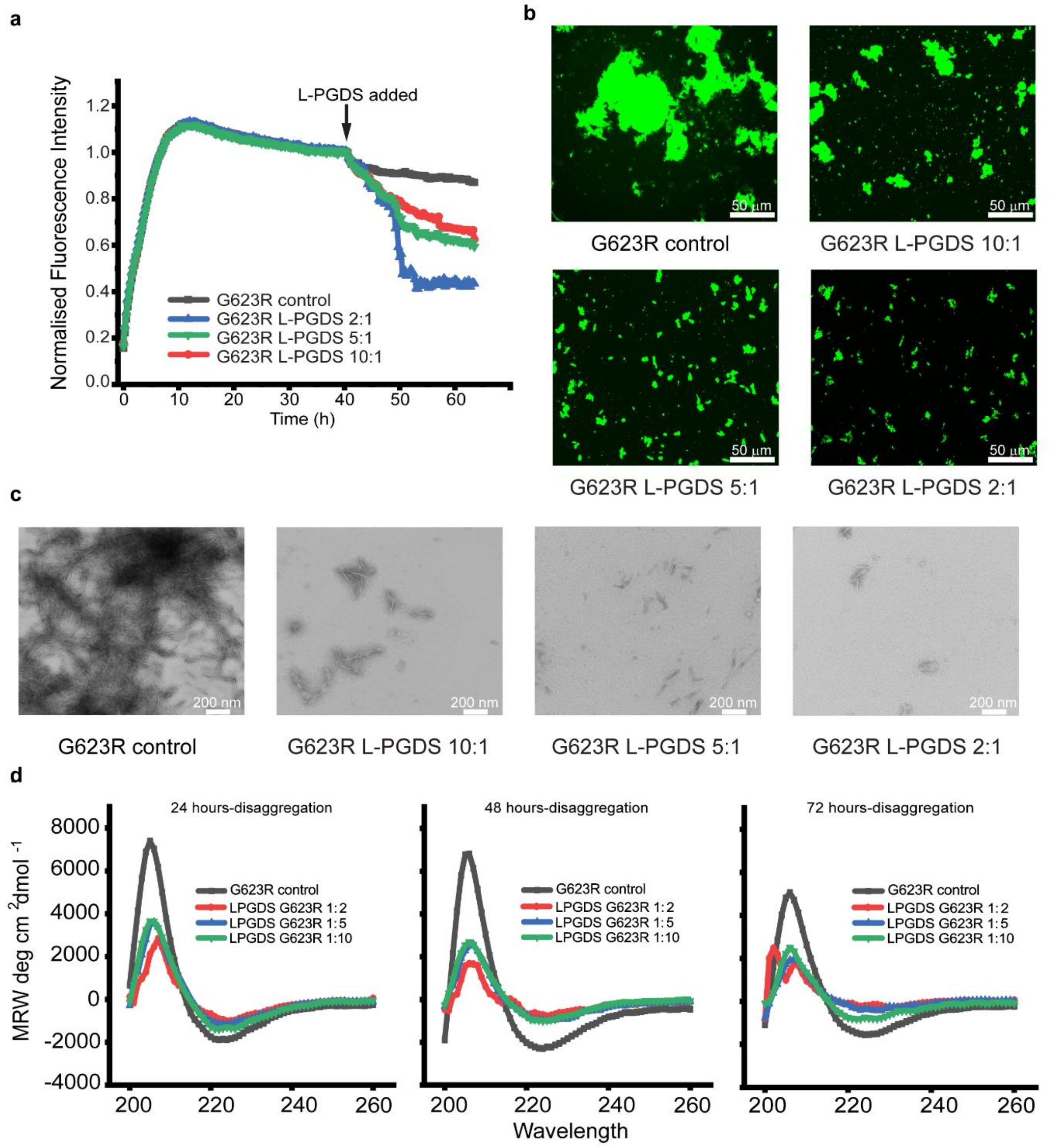

Figure S17: The amyloid disaggregation effects of L-PGDS on preformed G623R fibrils. **a** Long hour kinetic ThT assays. **b** Representative fluorescence microscopy images showing the amyloid disaggregation effects of L-PGDS on G623R fibrils after 72h incubation. **c** Representative negatively stained EM images showing the amyloid disaggregation effects of L-PGDS on G623R fibrils after 72h incubation. **d** Circular Dichroism spectra showing the fibril disaggregation effects of L-PGDS on preformed G623R fibrils.

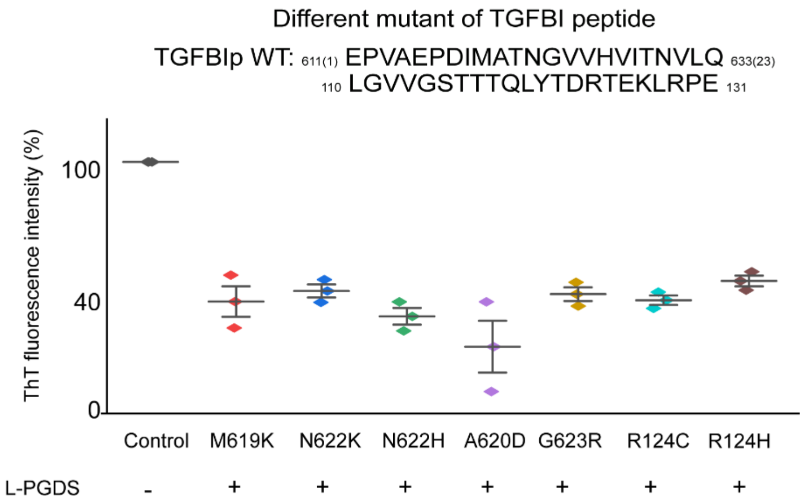

Figure S18: Amyloid disaggregation effects of L-PGDS for the sequence variants of TGFBIp associated with amyloid type corneal distrophy phenotype[3]. The ThT fluoresence intensity is normalised to the ThT fluorescence of the fibril control for each mutant. The WT TGFBIp sequence of the 611-633 and 110-131 regions are listed on top of the graph. Number of experiment(n) = 1 with triplicates for each condition and S.E.M calculated for the triplicates.

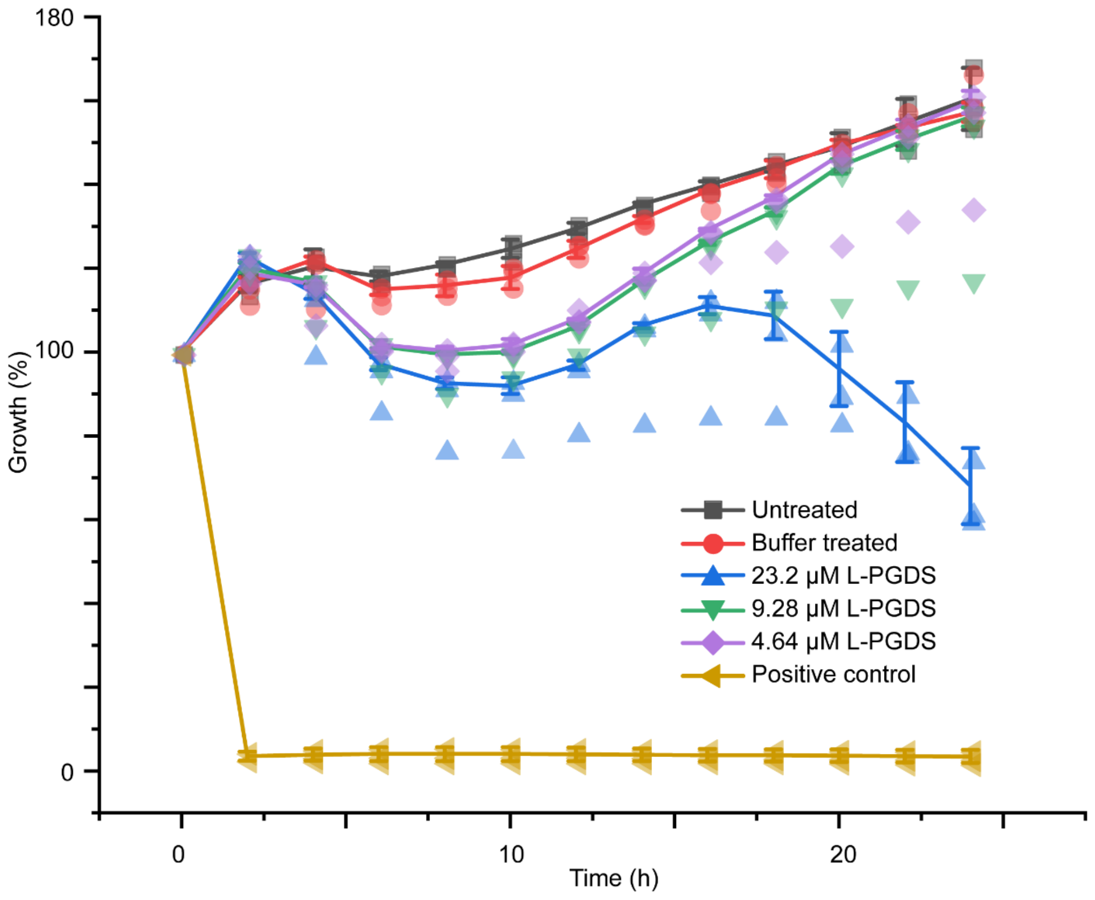

Figure S19: Cytotoxicity of WT L-PGDS at different concentrations, buffer only, untreated (UT) and positive control (DMSO) on cultured human corneal fibroblasts. Number of

experiment(n) = 1 with triplicates for each condition and S.E.M calculated for the triplicates.

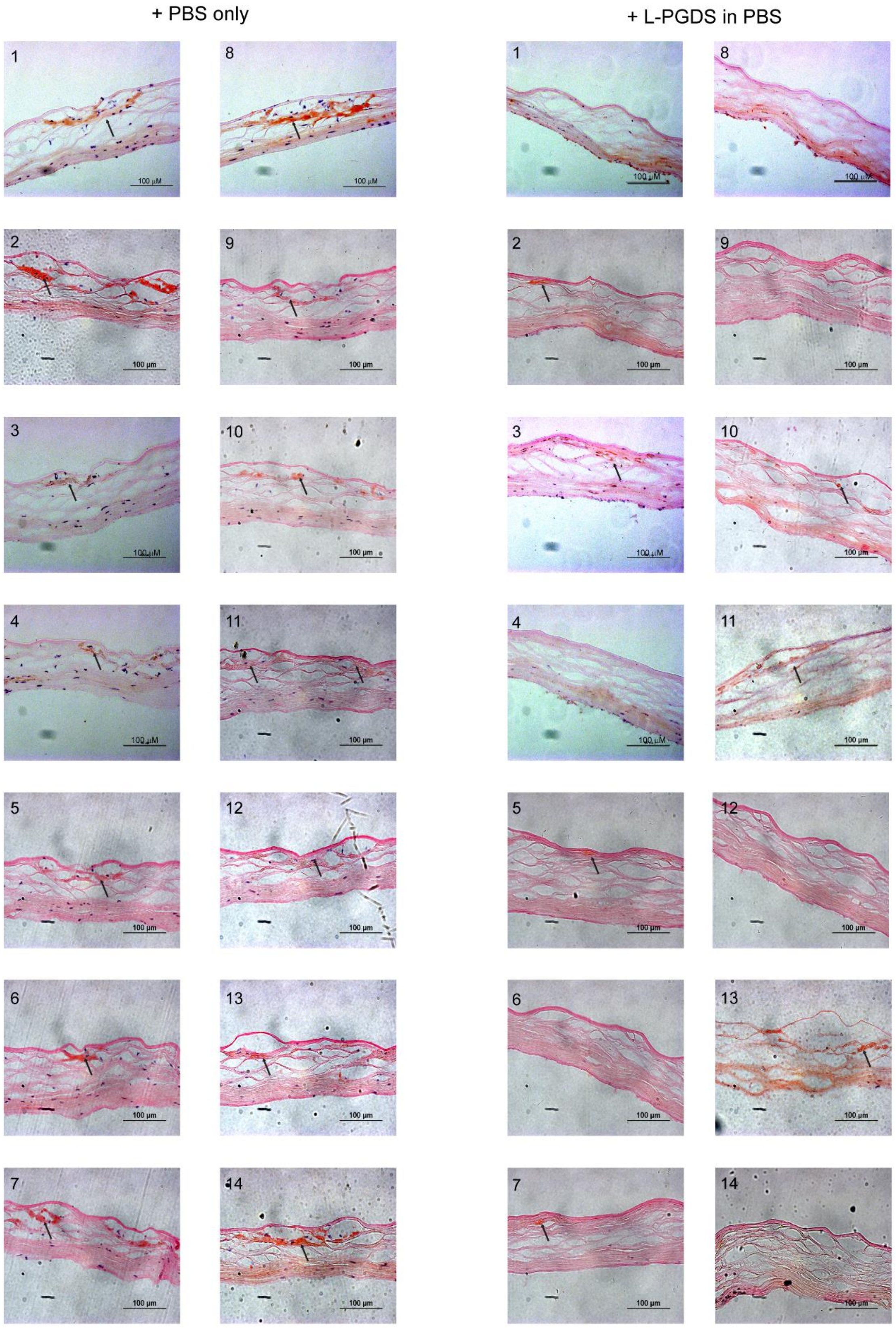

Figure S20: Corneal images collected from a patient with R124C mutation with Congo red staining incubated with control PBS buffer and the same buffer containing 25 µM L-PGDS. The amyloid deposits are indicated by the black arrow.

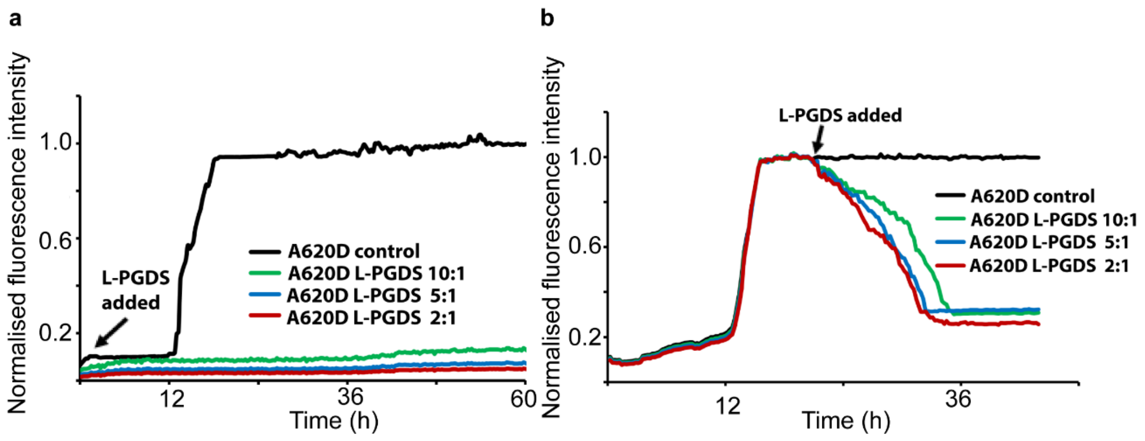

Figure S21: ThT fluorescence assays showing the **a** inhibitory and **b** disaggregation effects of L-PGDS on A620D mutant peptide and preformed fibrils respectively.

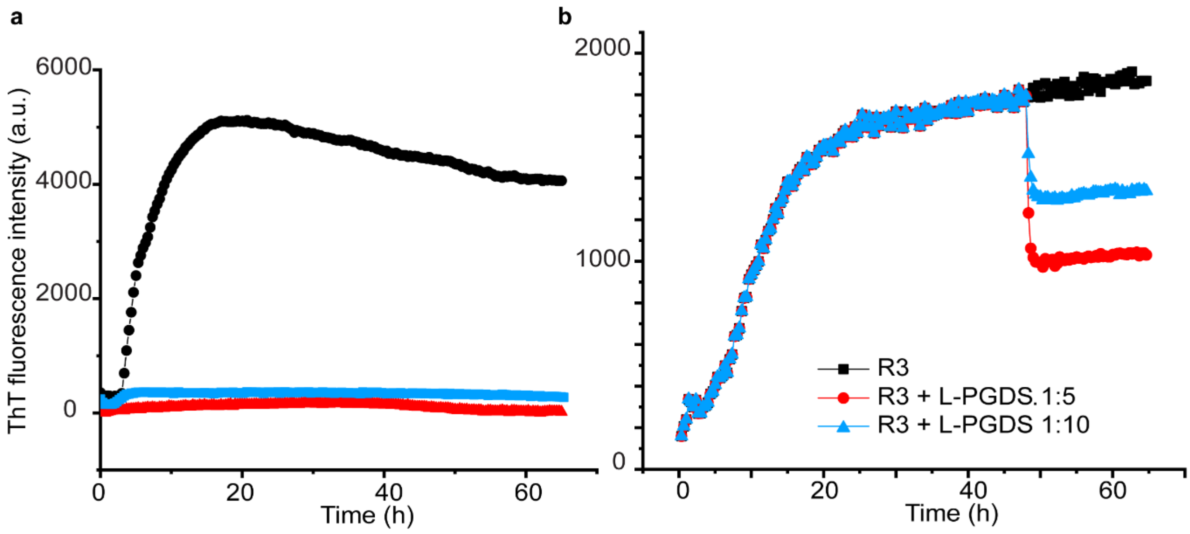

Figure S22: ThT fluorescence assays showing **a** the inhibition and **b** disaggregation effects of L-PGDS on Tau R3 preformed fibrils (Tau R3 peptide sequence: VQIVYKPVDLSKVTSKCGSLGNIHHK)

Supplementary table:

Table S1: ^{13}C and ^{15}N NMR chemical shift values (ppm) for ^{13}C- and ^{15}N-labeled sites in G623R fibrils, referenced using the DSS scale with adamantane as a secondary standard for ^{13}C and calculated indirectly for ^{15}N. Double peaks observed in extended or turn conformation are labelled as E and T, respectively. (Chemical shift perturbation of respective amino acids after the addition of L-PGDS)

Residue	CA	N	C	CB	CD1/CD2	CG1	CG2	CZ	CE
**E611**	54.4<br>(E/T:53.5)		177.9	29.6<br>(E/T:31.5)	180.6	36.2<br>(E/T:35.1)			
**P612**	60.8		175.1	E:34.3<br>T:33.9		25.6			
**V613**	61.2<br>(T:60.2)	E:127.2<br>T:122.7	175.8	E:36.4<br>T:37.0<br>(T:38.4)		22.8	21.6		
**A614**	54.2<br>(T:54.8)	E:128.1<br>T:124.6	176.2	E:22.5<br>T:23.1<br>(T:22.9)					
**E615**	55.6	E:110.0<br>T:107.4	178.1<br>(T:177.5)	E:30.4<br>T:29.6<br>(T:30.3)	E:180.5<br>T:183.3<br>(T:183.6)	E:36.3<br>T:36.0<br>(T:37.0)			
**P616**	E:62.1<br>T:63		173.8	E:32.9<br>T:32.3		27.3			
**D617**	52.3<br>(E:53.2)<br>(T:50.5)	E:126.6<br>T:130.1	174.0	E:41.0<br>T:41.8<br>(E:40.8)<br>(T:41.3)		E:176.4<br>T:175.3			
**I618**	60.5<br>(E/T:59.7)	121.5	173.7	36.7<br>(E/T:37.1)	12.7<br>(E/T:15.0)	26.9	17.0		
**M619**	54.9	E:129.0<br>T:131.3	173.8	E:33.1<br>T:31.1		30.4			
**A620**	55.2	127.7	173.8						
**T621**	61.7	122.5	172.4	70.1			21.5		
**N622**	53.2	127.0	174.0	43.1		175.0			
**R623**	55.6	E:130.5<br>T:126.7	175.2<br>(T:173.5)	30.8<br>(T:29.3)	E:42.1<br>T:41.6<br>(T:41.1)	27.8		159.1<br>159.7<br>(E/T:164)	
**V624**	61.5	125.5	175.3	35.8		22.8	20.5		
**V625**	61.4	126.8	175.4	36.0		23.0	20.6		
**H626**	52.8	125.8	174.1<br>(E/T:173.5)	34.4<br>(E/T:35.1)	117.9	137.4<br>(E/T:136.5)			139.0<br>(136.8)
**V627**	61.2	E:125.2<br>T:130.5	175.7	33.5		21.0	20.0		
**I628**	59.6	130.6	175.2	39.4	13.4	27.0	17.0		
**T629**	58.5	108.3	173.8	71.2			22.5		
**N630**	55.5	121.2	174.2	36.9		175.9			
**V631**	62.0	124.9	175.5	33.6		21.0	20.1		
**L632**	55.7	128.2	177.8	42.1	CD1: 26.0<br>CD2: 25.1	27.8			
**Q633**	55.7		175.8	31.2	180.4	34.1			

Table S2: Chemical shift prediction by online server, Proshift[4].

Residue No.	Amino acid	Atom	Extended conformation	Turn conformation
611	E	HA	4.35	4.35
611	E	C	176.6	176.6
611	E	CA	56.4	56.4
611	E	CB	29.7	29.7
612	P	HA	4.674	4.492
612	P	C	176.495	176.161
612	P	CA	62.854	63.155
612	P	CB	30.831	30.45
613	V	N	121.643	115.776
613	V	HA	4.188	4.386
613	V	C	175.561	175.298
613	V	CA	62.148	61.85
613	V	CB	32.542	32.978
613	V	HN	7.903	7.87
614	A	N	129.827	126.849
614	A	HA	4.508	4.416
614	A	C	176.399	176.736
614	A	CA	51.541	51.457
614	A	CB	19.99	19.832
614	A	HN	8.238	8.012
615	E	N	122.366	120.605
615	E	HA	4.589	4.42
615	E	C	175.507	175.9
615	E	CA	55.327	55.551
615	E	CB	30.515	29.816
615	E	HN	8.898	8.316
616	P	HA	4.581	4.422
616	P	C	175.749	175.779
616	P	CA	62.696	63.345
616	P	CB	32.358	30.56
617	D	N	118.497	121.935
617	D	HA	4.727	4.581
617	D	C	175.364	175.585
617	D	CA	53.691	53.609
617	D	CB	42.62	44.005
617	D	HN	8.318	7.996
618	I	N	125.013	126.252
618	I	HA	4.372	4.068
618	I	C	175.676	175.386
618	I	CA	61.06	62.265
618	I	CB	37.752	38.025
618	I	HN	8.403	8.422
619	M	N	127.468	130.679

619	M	HA	4.768	4.728
619	M	C	174.842	174.883
619	M	CA	54.924	55.008
619	M	CB	34.56	32.245
619	M	HN	8.444	8.65
620	A	N	125.329	126.208
620	A	HA	4.627	4.789
620	A	C	176.774	176.905
620	A	CA	51.641	52.102
620	A	CB	20.443	19.648
620	A	HN	8.372	8.445
621	T	N	118.867	119.861
621	T	HA	4.348	4.489
621	T	C	173.822	173.258
621	T	CA	62.359	62.199
621	T	CB	69.217	68.882
621	T	HN	8.342	8.528
622	N	N	123.59	121.77
622	N	HA	4.864	4.954
622	N	C	174.38	174.457
622	N	CA	52.994	52.765
622	N	CB	39.602	39.776
622	N	HN	8.604	8.419
623	R	N	125.934	121.957
623	R	HA	4.554	4.62
623	R	C	174.839	175.353
623	R	CA	55.91	55.796
623	R	CB	31.147	31.452
623	R	HN	8.557	8.368
624	V	N	123.855	122.462
624	V	HA	4.468	4.387
624	V	C	175.149	175.834
624	V	CA	61.86	62.054
624	V	CB	32.958	33.088
624	V	HN	8.43	8.353
625	V	N	125.783	126.315
625	V	HA	4.409	4.34
625	V	C	174.359	175.068
625	V	CA	61.556	62.143
625	V	CB	33.556	32.222
625	V	HN	8.314	8.388
626	H	N	126.049	125.861
626	H	HA	4.923	4.724
626	H	C	173.197	174.767
626	H	CA	55.314	55.779
626	H	CB	32.241	29.563

626	H	HN	8.217	8.414
627	V	N	122.794	129.62
627	V	HA	4.249	4.209
627	V	C	174.599	174.846
627	V	CA	61.933	61.537
627	V	CB	34.133	32.231
627	V	HN	8.318	8.643
628	I	N	127.414	127.797
628	I	HA	4.496	4.41
628	I	C	175.325	175.905
628	I	CA	60.895	60.743
628	I	CB	38.045	37.815
628	I	HN	8.473	8.276
629	T	N	119.695	120.452
629	T	HA	4.612	4.6
629	T	C	174.586	174.257
629	T	CA	61.445	61.264
629	T	CB	69.586	69.972
629	T	HN	8.392	8.495
630	N	N	124.446	122.923
630	N	HA	4.839	4.767
630	N	C	174.313	175.475
630	N	CA	52.977	52.75
630	N	CB	39.697	40.007
630	N	HN	8.812	8.633
631	V	N	122.412	121.858
631	V	HA	4.369	3.933
631	V	C	175.336	176.439
631	V	CA	61.909	62.679
631	V	CB	33.146	32.88
631	V	HN	8.394	8.294
632	L	N	129.012	128.038
632	L	HA	4.353	4.13
632	L	C	177.69	177.72
632	L	CA	55.42	56.115
632	L	CB	42.293	41.719
632	L	HN	8.516	8.332
633	Q	N	119.801	119.801
633	Q	HA	4.341	4.341
633	Q	CA	55.804	55.804
633	Q	CB	28.739	28.739
633	Q	HN	8.228	8.271

Table S3: Restraints used in G623R fibril structural calculation with CYANA.

	^{13}C-^{13}CDipole coupling (long range contacts)		Torsion angle	
**Residues**	**Inter residues (unambiguous)**	**Low ambiguity**	**Phi**	**Psi**
**E611**		E611C-E611CG	-85.4	112.7
**P612**	P612CB-M619CB<br>P612CB-D617CB			
**V613**	V613CB-M619CB<br>V613CG2-E615C<br>V613CB-R623CA		-146.7	122.5
**A614**	A614CB-D617CG<br>A614CB-P616CA		-180.0	134.4
**E615**	E615CD-R623CB	E615CG-A614C	-85.6	112.6
**P616**				
**D617**	D617CB-V614CG2<br>D617CA-I618CG2<br>D617CB-I618C<br>D617CB-V615C<br>D617CA-I618CG2		-109.6	30.6
**I618**	I618CB-V627CG1<br>I618C-M619CB<br>I618C-V625C<br>I618CG1-V625CB		-118.2	102.4
**M619**			-130.7	109.2
**A620**			-145.8	111.6
**T621**	T621CA-N622C<br>T621CB-R623CZ<br>T621CB-V624C<br>T621CB-I628CA		-135.5	111.9
**N622**			-145.8	113.0
**R623**	R623CZ-E615CD<br>R623CZ-D617C<br>R623CA-V615CB		-125.4	107.7

	R623CA-V615CB<br>R623CD-V625CB			
**V624**	V624CB-R623CA		-136.3	110.5
**V625**			-139.3	108.4
**H626**	H626CB-N630CA<br>H626CB-N630CG	H626CA-V627CA<br>H626CG-I628CB<br>H626CG-I628CG	-142.1	111.4
**V627**		V627CG1-I628CA<br>V627CA-I628CA	-140.0	103.8
**I628**		I628CD1-V627CA	-134.4	107.6
**T629**	T629CB-Q633C	T629CG2-N630CA<br>T629CG2-N630CB<br>T629CA-N630CG	-159.6	131.0
**N630**	N630CB-Q633CD		-94.4	110.5
**V631**			-144.7	99.4
**L632**	L632CD1-Q633CB	L632CB-Q633CB	-95.3	-50.5
**Q633**	Q633CD-N622CG			

Table S4: Frustration levels from residues 611-617 calculated by online server, Frustratometer[2].

Residues pair	Frustration level in TGFBI G623R fibril				Frustration level in TGFBI G623R fibril L-PGDS complex			
	Frustration state	Frustration energy (kcal/mol)	Frustration index	Well state	Frustration state	Frustration energy (kcal/mol)	Frustration index	Well state
617ASP$_{N}$-617ASP$_{N+1}$ (inter)	Highly	1.284	-2.947	Short	-	-	-	-
617ASP$_{N}$-617ASP$_{N-1}$ (inter)	Highly	1.034	-2.375	Short	-	-	-	-
617ASP$_{N}$-619MET$_{N}$ (intra)	Highly	0.657	-1.508	Short	Neutral	-0.033	0.074	Water-mediated
617ASP$_{N}$-612PRO$_{N}$ (intra)	Highly	0.893	-2.051	Short	Neutral	-0.147	0.333	Water-mediated
617ASP$_{N}$-614ALA$_{N+1}$ (inter)	Highly	0.821	-1.885	Short	-	-	-	-
617ASP$_{N}$-614ALA$_{N}$ (intra)	Highly	0.516	-1.185	Short	-	-	-	-
617ASP$_{N}$-615GLU$_{N}$ (intra)	Highly	0.63	-1.447	Long	Neutral	-0.142	0.322	Water-mediated
617ASP$_{N}$-612PRO$_{N-1}$ (inter)	-	-	-	-	Highly	0.615	-1.398	Short
616PRO$_{N}$-614ALA$_{N+1}$	Highly	0.919	-2.109	Short	Highly	0.467	-1.062	Short
616PRO$_{N}$-616PRO$_{N+1}$ (inter)	Highly	0.734	-1.686	Short	-	-	-	-
616PRO$_{N}$-616PRO$_{N-1}$ (inter)	Highly	0.54	-1.24	Short	Minimally	-0.504	1.145	Water-mediated
615GLU$_{N}$-612PRO$_{N+1}$ (inter)	-	-	-	-	Highly	0.685	-1.557	Short
615GLU$_{N}$-612PRO$_{N}$ (intra)	-	-	-	-	Highly	1.064	-2.418	Short
615GLU$_{N}$-615GLU$_{N+1}$ (inter)	Highly	1.215	-2.789	Short	Neutral	0.081	-0.184	Water-mediated
615GLU$_{N}$-615GLU$_{N-1}$ (inter)	Highly	1.174	-2.695	Short	Neutral	0.124	-0.281	Water-mediated
615GLU$_{N}$-614ALA$_{N+1}$ (inter)	Highly	0.653	-1.498	Short	Neutral	-0.018	0.041	Water-mediated
614ALA$_{N}$-611GLU$_{N}$ (intra)	-	-	-	-	Highly	0.764	-1.735	Short
614ALA$_{N}$-612PRO$_{N+1}$ (inter)	-	-	-	-	Highly	0.57	-1.296	Short
613VAL$_{N}$-611GLU$_{N}$ (intra)	-	-	-	-	Highly	1.248	-2.835	Short
613VAL$_{N}$-612PRO$_{N+1}$ (inter)	-	-	-	-	Highly	0.591	-1.344	Long
612PRO$_{N}$-619MET$_{N-1}$ (inter)	Highly	0.514	-1.181	Short	-	-	-	-
611GLU$_{N}$-613VAL$_{N+1}$ (inter)	Highly	0.537	-1.233	Short	-	-	-	-
611GLU$_{N}$-611GLU$_{N+1}$ (inter)	Highly	1.521	-3.493	Short	-	-	-	-
611GLU$_{N}$-611GLU$_{N-1}$ (inter)	Highly	1.459	-3.349	Short	-	-	-	-
611GLU$_{N}$-OPP 611GLU$_{N}$ (inter)	Highly	1.476	-3.39	Short	-	-	-	-
611GLU$_{N}$-OPP 611GLU$_{N-1}$ (inter)	Highly	1.456	-3.334	Short	-	-	-	-
Frustration free energy (kcal/mol/monomer)	5.34				1.585			
Frustration free energy released (kcal/mol/monomer)	-3.76							
Number of highly frustrated residue pairs from residues 611 to 617	19				8			
Total number of frustrated residue pairs	420				221			

Mean decoy energy for G623R fibril = -1.753 kcal/mol/monomer and Mean decoy energy for G623R/L-PGDS complex = -1.739 kcal/mol/monomer

***The units kcal/mol/chain and kcal/mol/monomer are equivalent and used interchangeably.**

Electrostatic interactions are highlighted in green.

Table S5: List of proteins that are enriched in amyloid deposits in corneal tissues[5] and released by PBS/L-PGDS treatment.

Protein	Quantity of protein released by PBS buffer	Quantity of protein released by L-PGDS	Fold Increase
Histone H2B type 1	371686.9375	7001935.5	18.8
E3 ubiquitin	96305.42188	690102.625	7.17
Tubulin beta-4B chain	624293.5	3168117.5	5.07
Complement component C8 gamma chain	139084.3438	570552	4.10
Vitronectin	29101.73828	72839.58594	2.50
Sushi repeat-containing protein SRPX2	40251.14844	81905.25781	2.03
C-type lectin domain family 11 member A	1072164.75	1911336.125	1.78
Heat shock protein beta-1	4898013.5	8259086.5	1.68
Serum amyloid P-component	410277.8125	638196.5	1.56
Alpha-enolase	13605672	20922466	1.54
Clusterin	3787982	5671733.5	1.50
Angiopoietin-related protein 7	3105031.5	4394892	1.42
Fibulin-1	330578.4688	451854.2188	1.37
Peroxiredoxin-1	9459418	12074252	1.28
Transforming growth factor-beta-induced protein ig-h3	97707320	120308304	1.23
Fibulin-5	82386.02344	94833.89844	1.15
Apolipoprotein A-IV	309395.8125	350152.2813	1.13
Serine protease HTRA1	95254.125	105348.8203	1.11

Table S6: Acquisition and processing parameters of ssNMR experiments used for resonance assignment of G623R fibrils and L-PGDS/G623R fibril complex

Experiment	NCACX	NCOCX	CANCO	NCA	NCO	CC	CC
Field (T)	18.8						
MAS rate (kHz)	17.857						
Probe	Bruker 1.9 mm HCN MAS probe						
Transfer 1	HN CP	HN CP	HC CP	HC CP	HC CP	HC CP	HC CP
Transfer time	1.2	1.2	1.4	1.2	1.2	1.4	1.4
Carrier (ppm) 13C, 15N	100	100	100	52	175	100	100
Transfer 2	NCA specific	NCO specific	CAN specific	NCA specific	NCO specific	DARR	DARR
Transfer time	3	3.5	3	3	3	20	75
Carrier (ppm) 13C, 15N	100	100	119	115	115	100	100
Transfer 3	DARR	DARR	NCO specific	-	-	-	-
Transfer time	75	75	4.5	-	-	-	-
Carrier (ppm) 13C, 15N	119	119	100	-	-	-	-
Digitalisation, F1	N	N	CA	N	N	C	C
T1 increments	36	36	72	224	224	896	896

Sweep width (kHz)	2551	2551	5952.333	8928.5	8928.5	53571	53571
Acquisition time (ms)	7.05	7.05	6.04	12.5	12.5	8.3	8.3
Digitalisation, F2	CA	CO	N	C	C	C	C
T2 increments	78	30	36	1536	512	1664	1664
Sweep width (kHz)	5952.333	2231.75	2551	53571.43	17857.143	53571.43	53571.43
Acquisition time (ms)	6.55	6.72	7.05	14.3	14.3	15.5	15.5
Digitalisation, F3	C	C	C	-	-	-	-
T3 increments	1536	1536	1536	-	-	-	-
Sweep width (kHz)	53571.43	53571.43	53571.43	-	-	-	-
Acquisition time (ms)	14.3	14.3	14.3	-	-	-	-
Total scans	48	96	32	64	64	16	16
Recycle delay	1.5	1.5	1.5	1.5	1.5	1.5	1.5
Total experiment time	63h	48h	40h	6h	6h	6h 20 min	6h 33 min
Window function (WDW)	QSINE	QSINE	QSINE	QSINE	QSINE	QSINE	QSINE
Sine bell shift (SSB)	0.1	0.1	0.1	0.1	0.1	0.1	0.1

Supplementary References

1 Usov, I. & Mezzenga, R. FiberApp: An Open-Source Software for Tracking and Analyzing Polymers, Filaments, Biomacromolecules, and Fibrous Objects. *Macromolecules* **48**, 1269-1280, doi:10.1021/ma502264c (2015).

2 Parra, R. G. *et al.* Protein Frustratometer 2: a tool to localize energetic frustration in protein molecules, now with electrostatics. *Nucleic acids research* **44**, W356-360, doi:10.1093/nar/gkw304 (2016).

3 Anandalakshmi, V. *et al.* Effect of position-specific single-point mutations and biophysical characterization of amyloidogenic peptide fragments identified from lattice corneal dystrophy patients. *The Biochemical journal* **474**, 1705-1725, doi:10.1042/BCJ20170125 (2017).

4 Meiler, J. PROSHIFT: protein chemical shift prediction using artificial neural networks. *J Biomol NMR* **26**, 25-37, doi:10.1023/a:1023060720156 (2003).

5 Venkatraman, A. *et al.* Proteomic Analysis of Amyloid Corneal Aggregates from TGFBI-H626R Lattice Corneal Dystrophy Patient Implicates Serine-Protease HTRA1 in Mutation-Specific Pathogenesis of TGFBIp. *J Proteome Res* **16**, 2899-2913, doi:10.1021/acs.jproteome.7b00188 (2017).
