# Supplementary material for: Release of frustration drives corneal amyloid disaggregation by brain chaperone
Source: Commun Biol. 2023 Mar 30;6:348. doi: 10.1038/s42003-023-04725-1 (PMC10063603; doi:10.1038/s42003-023-04725-1)
Supplement: Supplementary file 2 — Description of Additional Supplementary Files [file 42003_2023_4725_MOESM2_ESM.pdf]

Description of Additional Supplementary Files

File name: Supplementary Data 1

Description: The source data behind the graphs in the paper.

File name: Supplementary Data 2

Description: Initial coordinate of MD simulation

File name: Supplementary Data 3

Description: MD Coordinate file for Figure S14

File name: Supplementary Data 4

Description: MD simulation input file

File name: Supplementary Video

Description: MD simulation video of the TGFBIp G623R fibril and L-PGDS complex.
